# A systematic, integrative review exploring supports that promote the retention of employees working in the aged care sector

**DOI:** 10.1111/ajag.70070

**Published:** 2025-07-31

**Authors:** Britt O'Keefe, Eva Yuen, Susan Perlen, Alison M. Hutchinson

**Affiliations:** ^1^ School of Nursing and Midwifery, Centre for Quality and Patient Safety Research (QPS) in the Institute for Health Transformation Geelong Victoria Australia; ^2^ Monash Health Clayton Victoria Australia; ^3^ Barwon Health Geelong Victoria Australia; ^4^ Faculty of Health Sciences University of Southern Denmark Odense Denmark

**Keywords:** employee turnover, health personnel, homes for the aged, review

## Abstract

**Objective:**

To systematically synthesise existing literature and identify supports to promote employee retention in the aged care sector.

**Methods:**

A systematic integrative review was conducted following Whittemore and Knafl's (2005) methodology framework and guided by the Preferred Reporting Items for Systematic Reviews and Meta‐Analyses guidelines. A comprehensive search for studies published between 1997 and 2024 was undertaken across Business Source Complete, CINAHL Complete, Medline Complete and APA PsycInfo (via administration, pastoral and ancillary care workers influencing employee retention in the aged care sector were included), with qualitative findings and statistically significant quantitative data extracted. Methodological quality was assessed using the Mixed Methods Appraisal Tool, and the data were analysed inductively using a thematic approach.

**Results:**

Eighteen studies were included in the review, comprising 13 quantitative, four qualitative and one mixed‐methods study. The factors and strategies were themed into six areas relating to the retention of the aged care workforce: (1) employee characteristics, stability and well‐being, (2) workplace relationships, (3) training opportunities and career growth, (4) recognition, incentive and compensation, (5) organisational structure, culture and resources and (6) value‐driven and empowered care practice.

**Conclusions:**

This review identified employee, employer and organisational factors and strategies that influence workforce retention within the aged care sector. While there is a paucity of evidence available, these findings are a critical first step to guide further design, implementation and evaluation of retention strategies to address the challenges of workforce retention in the aged care sector.

**Practice impact**

Strategies are urgently needed to ensure workforce retention meets the growing demands of an ageing population. Despite the limited evidence on retention supports for aged care workers, this review identified factors and strategies that can be applied by aged care operators to help achieve a stable and competent workforce.

**Policy impact**

The findings highlight the need for long‐term strategic workforce planning to address the shortage of direct care workers within Australia's aged care sector, and increased funding for evidence‐informed retention initiatives. As demand for aged care services increases, policies must prioritise workforce stability, mitigating the projected direct care workforce shortages.

## 1 INTRODUCTION

High‐income countries are experiencing unprecedented increases in their populations of older people.^1^, ^2^ In Australia, life expectancy has steadily increased since 1980^3^ and the number of Australians aged 85 years and over is projected to exceed 1.5 million by 2058, constituting 4% of the population.^3^, ^4^ This demographic shift, coupled with an increase in chronic health conditions, has driven rising demand for aged care services and supports.^1^, ^3^, ^4^ According to the Productivity Commission, by 2050 more than 3.5 million Australians are projected to require aged care services.^5^

Australia's aged care services cover the continuum of care, including (1) the Commonwealth Home Support Program (CHSP) offering entry‐level home care support, (2) the Home Care Packages Program (HCP) for people with more complex home care needs and (3) residential care, encompassing both permanent and respite care.^6^ The workforce for these services is extensive and diverse, comprising approximately 420,000 individuals,^7^ including health professionals, support staff and ancillary staff, along with management and administrative support personnel. The occupational groups include registered nurses (RN), enrolled nurses (EN), nurse practitioners, allied health professionals, personal care workers (PCW), community care workers, administration staff, pastoral and ancillary care workers.^8^ Globally, occupational titles for PCWs vary, including certified nursing assistants, nursing aides and personal carers, despite shared responsibilities. For consistency, we use the term personal care workers (PCW) throughout this review.

Despite the increased demand for aged care services, aged care operators grapple with managing the current rate of turnover in the workforce, particularly the direct care workforce.^9^ The direct care workforce in Australia provides care directly to care recipients as a core component of their work and is comprised of RNs, ENs, PCWs and allied health professionals.^8^ Anticipating the growth in Australia's ageing population, a fourfold increase in the aged care workforce has been projected, requiring an additional 830,000 to 1.3 million direct care workers by 2050.^4^, ^10^, ^11^ From 2020, the CHSP program employed 76,096 workers (59,029 in direct care), the HCP program employed 80,340 workers (64,019 in direct care), and the residential aged care sector employed 277,671 workers (208,903 in direct care).^8^ This represents a 32% increase in full‐time equivalent positions since 2016.^8^ In the absence of proactive employee retention measures, a shortfall is anticipated to escalate to 400,000 direct care workers by 2050.^7^

Retention in the context of employment refers to the duration of time in which an employee remains within an organisation.^12^ This has emerged as a major concern among aged care operators, particularly for managing workforce shortages. Such shortages are exacerbated by high turnover and low retention rates across the aged care workforce. According to the latest published data from November 2019 to November 2020, turnover rates were 37% for RNs, 28% for ENs, 28% for PCWs, 25% for allied health professionals and 28% for allied health assistants.^8^ In contrast, the overall job mobility rate (changing industry or occupation, job tenure or changes in current job) in Australia was 8% in 2024.^13^ Although these metrics capture distinct facets of workforce mobility and pertain to different periods of time, they highlight the comparatively high rate of turnover in the aged care sector relative to general employee mobility across Australia. Understanding, implementing and evaluating factors and strategies for retaining aged care workers is critical for aged care operators to promote employee retention and lower the high turnover rates. Such measures are essential to ensuring a stable and competent workforce capable of meeting the growing needs of an ageing population,^14^ while reducing operating costs and improving employee well‐being.

The aim of this integrative review was to synthesise literature that investigated supports to promote employee retention in the aged care sector. The research question was as follows: What retention supports are effective and not effective in reducing the turnover of employees working in the aged care sector? For the purposes of this study, supports included factors and strategies that were found to be effective and not effective in reducing turnover. This review encompassed studies addressing the entire aged care workforce across home care, assisted living and residential aged care.

## 2 METHODS

### 2.1 Study design

A systematic integrative review was undertaken to capture articles using quantitative, qualitative and mixed‐methods research methodologies that identified factors and strategies to promote the retention of employees working in the aged care sector. Whittemore and Knafl's^15^ integrative review process, comprising five stages to enhance rigour, was used to synthesise the evidence included in the review. Additionally, the Preferred Reporting Items for Systematic Reviews and Meta‐Analysis (PRISMA) statement and checklist^16^ guided the reporting of the review. The review protocol was prospectively registered on PROSPERO (https://www.crd.york.ac.uk/PROSPERO/), registration number CRD42022361696.

### 2.2 Search strategy

A systematic electronic literature search was developed in consultation with a research librarian. The search was conducted in August 2022 and updated in June 2024. Keywords and MesH terms relating to ‘employee retention’, ‘workforce’, ‘aged care’ and ‘retention strategies’ were used. The full search was performed using the EBSCOHost platform in the Business Source Complete, CINAHL Complete, MEDLINE Complete and APA PsycInfo databases and the Embase platform (excluding Medline) (Appendix S1). One author performed both searches.

### 2.3 Inclusion and exclusion criteria

A two‐stage screening process was conducted using Covidence. After removing duplicates, two authors independently reviewed the articles' titles and abstracts, applying the criteria in Table 1. Discrepancies in screening decisions were resolved through discussion. One author documented inclusion/exclusion reasons during the full‐text screening.

**TABLE 1:** Inclusion and exclusion criteria.

Inclusion criteria	Exclusion criteria
Peer‐reviewed articles reporting empirical studies	Reviews of literature, including systematic reviews, scoping reviews, integrative reviews, rapid reviews, umbrella reviews and literature reviews
Articles reporting quantitative, qualitative and mixed‐method studies	Conference proceedings, abstracts, dissertations, protocols, letters, editorials, commentaries and books
Articles published after 1 July 1997, to align with the marketisation of Australia's aged care sector following the passing of the *Aged Care Act 1997*, which allowed the establishment of private residential aged care operators, fundamentally reshaping the landscape of aged care and mirroring today's realities	Grey literature, including unpublished studies and reports
Articles published in English	
Articles reporting retention factors and strategies found to be effective in reducing turnover	
Articles reporting on employees who directly or indirectly provide support to older people in receipt of aged care services and supports	
Articles reporting studies conducted with older people in receipt of aged care services and supports delivered in their home or in a residential setting	

### 2.4 Quality appraisal

The methodological quality of the included studies was appraised independently by two authors using the Mixed Methods Appraisal Tool (MMAT) version 2018.^17^ The MMAT is designed for appraising the methodological quality of quantitative, qualitative and mixed‐methods studies in a single tool. Studies are assessed across five different categories based on the study design. Questions in each category are rated either ‘Yes’, ‘No’ or ‘Can't tell’. Based on the ratings, an overall score is calculated using a scale ranging from 1 to 5 (1 = low quality, 2 = average quality, 3 = good quality, 4 = high quality and 5 = excellent quality). Adhering to the guidelines established by Hong et al.,^17^ studies were retained regardless of their methodological quality scores. Discrepancies in the quality appraisal ratings were resolved through verbal discussions between the two authors until consensus was reached.

### 2.5 Data extraction and synthesis

Following Whittemore and Knafl's framework involving data reduction, data display, data comparison, conclusion drawing and verification,^15^ a standardised data extraction table was developed to capture data related to retention factors and strategies, facilitating a descriptive summary for each study. Quantitative data showing statistical significance, including descriptive statistics and *p*‐values, were recorded in the data extraction table and are detailed in Table 2. Qualitative data were extracted (Appendix S2) and thematically analysed, guided by Braun and Clarke's^18^ six‐step process. The extracted data were compared and contrasted repeatedly between the qualitative and quantitative findings to identify relationships. This process involved systematically integrating and refining the data to identify patterns, connections and relationships.^15^ The data extracted by one author were independently reviewed and verified for accuracy by another author using the primary source.^19^ The final set of themes and sub‐themes was iteratively refined by one author and collaboratively reviewed and discussed by all authors, with consensus reached through discussion and joint refinement (Table 3).

**TABLE 2:** Characteristics of included studies.

Author (year), country	Aim/objective	Study design, data collection	Setting, participant sample size, response rate & characteristics	Data collection methods	Data analysis	Results^a^/findings	MMAT score	Retention definition	Limitations
Berridge et al. (2018), USA	To examine the relationship between staff empowerment practices and CNA retention	Secondary analysis of cross‐sectional, survey	Setting: 4149 stratified selected NHs across USA Participation rate: not reported Participant sample: *n* = 2034 NHAs and DoNs Response rate: 63% (2215) *Participant characteristics*: Gender: unreported Age: unreported Tenure: unreported Race: unreported	Staff empowerment practices: seven items using never, sometimes, often and always scale Retention: single study‐developed item on NH administrator survey: ‘About what percent of the NURSING ASSISTANTS who were employed at your nursing home TODAY has worked at the nursing home for at least 12 months’, using four scales of 0%–50%, 51%–75%, 76%–90%, 91%–100% Secondary data – Online Survey Certification and Reporting, Area Resource File and Aggregated Minimum Data Set 2009	Ordered logistic regression models were used to evaluate associations between staff empowerment practice score quartiles and CNA retention and adjusted for covariates	A higher staff empowerment score at quartile 2 (AOR 1.44, CI 1.15, 1.80, *p* ≤ .01) and quartiles 3 and 4 (AOR 1.64, CI 1.34, 2.00, *p* ≤ .01) was positively associated with higher CNA retention when compared to quartile 1 Retention was also positively associated with NHs with lower NHA turnover, only one NHA in the past year (OR 1.77, CI 1.33, 2.35, *p* ≤ .01), NHs with two NHAs in the last year (OR 1.41, CI 1.03, 1.92, *p* < .05), higher occupancy rates (OR 1.25, CI 1.14, 1.37, *p* ≤ .01), RN hours per day per resident (OR 2.30, CI 1.59, 3.34, *p* ≤ .01) and CNA hours per day per resident (OR 1.11, CI 1.01, 1.22, *p* < .05) Conversely, retention was negatively associated with for‐profit NHs (OR .75, CI .62, .90, *p* ≤ .01)	4	No	Cross‐sectional design, which hinders the assessment of causal relationships and changes over time. Additionally, the possibility of preexisting differences in retention rates, potential resistance to change in nursing homes, and the need for further research to explore additional empowerment initiatives
Chao and Lu (2020), Taiwan	To examine the differences in the determinants of ITS (T1) and actual retention behaviour at follow‐up (T2) between younger and older NA in LTC facilities	Longitudinal, survey	Setting: 137 LTC facilities, stratified random sampling for administrative regions, types and bed capacities to align with the population distribution selected in Taiwan Participation rate: 94% (137/146) Participant sample: NAs, 643 in 146 facilities at T1, not reported and 595 in 137 facilities at T2 Response rate: not reported *Participant characteristics*: Gender: 78% female (<45 years (younger)), 87% female (>46 years (older)) Age (mean): 36.15 younger, 54.42 older Tenure: not reported Race: not reported	Personal characteristics: five items (age, sex, marital status, education, monthly salary) Organisational support: five item perceived organisational support instrument (Eisenberger et al., 1997) using 1–5 score (from 1 (strongly disagree) to 5 (strongly agree)) Work latitude: six item scale (Abrahan & Hansson, 1995), using 5‐point Likert scale Selection, optimisation and compensation strategies: 12 item scale (Baltes et al., 1995) Burnout: 22 item Maslach Burnout Inventory using 7‐point Likert scale Retention: single item ‘still in the job at T2 to reflect actual retention after 2 years’ (no/yes)	Two‐level generalised linear modelling at T2: examine the retention of NAs as a function of various factors including personal characteristics, organisational factors, selection, optimisation, and compensation strategies, as well as burnout at T1, and the retention of NAs at T2 Separate multilevel modelling analyses were conducted for younger NAs (<45 years old), older NAs (>45 years old). Similar analytical methods were applied to examine retention status at T2, utilising personal and organisational factors, selection, optimisation, compensation strategies, and burnout as predictors Multilevel generalised linear modelling was utilised to identify determinants of actual retention behaviour at follow‐up, treating retention status as a categorical measure	At T2, factors positively associated with retention include being married (OR 11.974, (2.640, 54.316), *p* ≤ .01), using optimisation strategies (OR 3.352, (1.171, 9.597), *p* ≤ .05), and having a high ITS (OR 1.901 (1.066, 3.411), *p* ≤ .05). Conversely, having a college or above education (OR .047, (.002, .968), *p* ≤ .05) and lower emotional exhaustion (OR .924, (.854, .996), *p* ≤ .05), were negatively associated with retention When comparing younger and older CNAs, the factors associated with retention exhibited differences. Marital status (OR 2.544, (1.357, 4.737), *p* ≤ .01) were positively associated with employee retention, while gender (OR .457, (.223, .935), *p* ≤ .05) was negatively associated For older NAs, marital status (OR 1.853, (1.010, 3.400), *p* ≤ .05), work latitude (OR 1.085, (1.000, 1.177), *p* ≤ .05), optimisation (OR 1.959, (1.278, 3.003), *p* ≤ .01), and depersonalisation (OR 1.142, (1.060, 1.230), *p* ≤ .001) was positively associated with employee retention, while education attainment (college and above) (OR .260, (.088, .775), *p* ≤ .05) and emotional exhaustion (OR .959, (.931, .987), *p* ≤ .01) was negatively associated	4	No	The longitudinal relationship, measured at only two timepoints, between organisational and personal factors at T1 and retention at T2 limited causal interpretation, potential measurement errors on some subscales of the brief questionnaire, and the absence of comprehensive assessments of institutional environments and work conditions for nursing assistants
Creapeau et al. (2022), USA	To investigate practices that may help retain CNAs and address the staffing challenges in LTC	Qualitative, semi‐structured interviews	Setting: 59 purposively selected LTCFs in Midwestern region of America Participation rate: not reported Participant sample: *n* = 413,295 CNAs, 59 DoN, 59 Administrators. **Participant characteristics**: *CNAs* Gender: 77% female. Age (years): 15% ≤22, 27% 23–30, 24% 31–40, 14% 41–50, 20% ≥51. Tenure (years): 43% <2, 19% 2–3, 17% 4–7, 15% 8–20, 6% ≥21 Race: unreported *DoN* Gender: 81% female. Age: not reported. Tenure (years): 44% <2, 15% 2–3, 14% 4–7, 19% 8–20, 8% ≥21 Race: not reported *Administrators* Gender: 63% female. Age: not reported. Tenure (years): 29% <2, 19% 2–3, 25% 4–7, 20% 8–20, 7% ≥21 Race: not reported	No explicit mention of an interview guide, however four questions were asked during semi‐structured interview, (1) ‘What do you think are the biggest staffing challenges for CNAs?’, (2) ‘What do you think are the root causes for your staffing challenges in the CNA position?’ (3) ‘What do you think are the top 3 things CNAs are most looking for from employers?’ and (4) ‘What are the top 5 most useful strategies, factors, programs, or practices you've seen positively impact retention of CNAs?’	Content analysis	Participants reached a consensus indicating that the three most effective retention strategies were appreciation, positive working relationships and wages	5	No	Potential biases in responses, as participants might have given socially desirable answers. The study focused only on CNAs, DoNs, and NH administrators, potentially overlooking other stakeholders' perspectives. Reliance on a single method of data collection, which may have restricted the depth of understanding. Additionally, there is a possibility of recall bias affecting the accuracy of participants' reflections on staffing challenges and retention strategies. Lack of information on the response rate, which could affect sample representativeness and short length of interview may have impacted on information gained
Dill et al. (2013), USA	To examine the relationship between job satisfaction, intention, and retention of NAs in NHs and the role that contingency factors play in employment intention and retention	Secondary data analysis of cross‐sectional, survey	Setting: 18 purposively and convenience selected NHs in a southern USA state Participation rate: 100% (*n* = 18) Participant sample: *n* = 315 NAs Response rate: mean 95% (*n* = 449) **Participant characteristics**: Gender: 95% female Age (mean): 37.7 Tenure (years/months): 4.1 Race (black): 50%	Retention: whether participants continued to hold their position or job 12 months after the survey was completed (yes = 1) Job satisfaction, job characteristics (such as workload, supervisor support, quality of care) contingency (such as being primary bed winner, receiving public assistance), individual characteristics, organisational characteristics and economic characteristics were also measured	Logistic regression models were used to analyse retention in the field of LTC	Using regression analysis, Model 5 found retention was positively associated with those who had health insurance (Coef. .77, SE .37, *p* ≤ .05) tenure in the job (Coef. .47, SE .18, *p* ≤ .05) and negatively associated with Being a breadwinner (Coef. −.92, SE .37, *p* ≤ .05), past health care experience (Coef. −.86, SE .35, *p* ≤ .05)	3	No	The study's absence of specific reasons for individual turnover, the inability to distinguish between voluntary and involuntary turnover, considerable missing data affecting variability, small sample that was not representative, potential sample bias as NAs were from organisations awaiting placement on a workforce development program waiting list, and data collection preceded the economic recession, indicating a necessity to investigate predictors of retention in shifting economic climates
Donoghue (2010), USA	To provide national estimates of turnover and retention for RN, LPNs and CNAs in NHs and to examine associations between management tenure, organisational characteristics, local economic conditions turnover and retention	Secondary analysis of cross‐section, survey	Setting: 1174 systematically selected NHs participated in USA nationwide survey, 2004 National Nursing Home Survey Participation rate: 81% (1174/1500) Participant sample: not reported Response rate: not reported *Participant characteristics*: Gender: not reported Age: not reported Tenure: not reported Race: not reported	Retention rate: the percentage of FTE staff employed for more than 1 year Management tenure: the total number of months DoNs and NHAs were employed in their current positions at the same facility Independent variables: the following variables were included to predict retention rates, hours per patient day, overtime, tenure, wages, total number of RN FTEs at Bedside, proprietary Status, membership status, per capita income, total number of NHs in the county and unemployment rate Secondary data – 2004 National Nursing Home Survey and Area Resource File (2005)	National estimates of turnover and retention rates in NHs using statistical weights provided by the National Center for Health Statistics Descriptive statistics were used to summarise and present the turnover and retention data for RNs, LPNs, and CNAs Ordinary least squares regression: forecast retention levels using the following variables, staffing variables, organisational characteristics, and local economic conditions. Statistical weights for facility size applied	For RNs, LPNs and CNAs employed for 12 months or more, longer DoN tenure and higher average occupancy in the NH were positively associated with higher retention For RNs employed for 12 months or more, longer DoN tenure (.12, SE .02, *p* < .001), higher RN hours per patient per day (7.41, SE 2.57, *p* < .01), higher CNA hourly starting wage (2.88, SE .95, *p* < .01) and higher average occupancy in the NH (26.6, SE 8.03, *p* < .01), were positively associated with higher retention. Conversely, higher number of LPN overtime shifts in the last week (−.35, SE .14, *p* < .05), was negatively associated with lower retention For LPNs employed for 12 months or more, longer DoN tenure (.09, SE .02, *p* < .001), higher CNA hourly starting wages (2.22, SE .81, *p* < .01), and higher average occupancy in the NH (2.32, SE 7.89, *p* < .05) were positively associated with higher retention. Conversely, lower LPN hourly starting wages (−2.08, SE .58 *p* < .001) was negatively associated with lower retention For CNAs employed for 12 months or more, longer DoN tenure (.06, SE .01, *p* < .001), RN hours per patient per day (8.20, SE 2.67, *p* < .01), higher average occupancy in the NH (14.31, SE 6.46, *p* < .05) and higher local unemployment rate (.97, SE .34, *p* < .01), were positively associated with higher retention. Conversely, a higher number of LPN hours per patient per day (−3.92, SE 1.69, *p* < .05), higher LPN overtime shifts in the last week (−.21, SE .09, *p* < .05) and for‐profit NHs (−4.34, SE 1.65, *p* < .01) were negatively associated with lower retention	5	Yes	Cross‐sectional design hinders causal inference, issues with missing data as multiple survey items are needed to determine retention rates, reliance on a short 3‐month timeframe, limited generalisability, potential biases, a narrow scope of variables, and the omission of certain external factors that could influence staff retention in NHs
Dreher et al. (2019), USA	To increase CNA retention through an evidence‐based education training program on compassion fatigue awareness and multiple self‐care strategies	Pre‐ and posttest survey	Setting: One not for profit, state selected NH in Southeast region of USA Participation rate: not reported Participant sample: *n* = 45 CNAs Response rate: 96% (45/47) *Participant characteristics*: Gender: 93% female Age (years): 2% – <20, 11% – 20–29, 20% – 30–39, 27% – 40–49, 29% – 50–59, 11% – >60 Tenure (years as CNA): 7% – <1, 22% – 1–5, 2% – 6–10, 11% – 11–15, 9% – 16–20, 49% – >20 Race: not reported	Participant demographics: seven items (age, gender, years of experience as a CNA, highest level of education, usual shift worked, hours worked per week, and employer) Compassion satisfaction and compassion fatigue: Professional Quality of Life Scale, 30 items using 5‐point Likert scale, with three subscales, compassion satisfaction, burnout, and secondary traumatic stress Measured at three time points: preintervention, 1‐ and 3‐month postintervention Post implementation survey: feedback on education program Retention: assessed at four time points, preintervention, at 1‐, 3‐, and 4‐month post‐intervention	Descriptive statistics to analyse participant demographics Multivariate analysis of variance was used to examine the effect of the intervention at three time points, while univariate analysis and non‐parametric tests (Kruskal‐Wallis) were employed to compare outcome differences in compassion satisfaction, burnout, and secondary traumatic stress and then these were analysed preintervention, 1‐ and 3‐month postintervention Retention data compared to CNA retention in previous year	No statistical evidence of significant differences in retention rates after the intervention, despite observing increased CNA retention and reduced reliance on supplemental agency staff. However, the retention rates were higher in all 4 months following the intervention compared to the same months in the previous year	3	No	Small sample size at each data collection point limits generalisability. Data confidentiality issues preventing matching of participants for repeated measures analysis and a reliance on independent tests instead of dependent samples. Data collection during seasonal holidays which could have influenced outcomes
Frank et al. (2006), Canada	To identify the issues related to LTC medical directors' satisfaction with their work and to get their opinions about recruitment and retention of LTC physicians	Cross‐sectional, questionnaire	Setting: purposively selected LTCs across Canada Participation rate: not reported Participant sample: *n* = 387 medical directors Response rate: 55% (387/705) *Participant characteristics*: Gender: 88% male Age (years): 1% – ≤35, 19% – 36–45, 34% – 46–55, 35% – 56–65, 12% – 66–81 Tenure (years in LTC practice): mean = 18.70 (SD 9.35) Race: not reported	Measures not reported Responses to open‐ and closed‐ended questions and to Likert‐type scales	Descriptive analysis: factors related to physician satisfaction, recruitment, and retention Percentages and frequencies using Student *t*‐test, *χ* ^2^ test and odds ratios	No statistically significant results on employee retention were reported, but identified ten strategies in rank order of importance: increase fee schedule (70%), offer on‐call stipend (58%), offer alternative funding for remuneration (38%), increase nursing staff (29%), increase links with other directors (17%), increased role of nurse practitioners (15%), increased availability of other professionals (15%), develop large on‐call groups (8%), offer university affiliation for physicians (6%), develop academic nursing homes (5%)	3	No	Challenges in identifying medical directors in the fragmented LTC sector, lack of a centralised LTC system, and incomplete database coverage, which could introduce selection bias. Respondents were predominantly from smaller centres, potentially limiting generalisability to urban or rural settings. The male‐dominated respondent pool may reflect practice pattern differences. Perspectives of medical directors, with their expanded roles, may not fully represent all LTC physicians, impacting the broader applicability of the findings
Hegeman et al. (2007), USA	To evaluate the effectiveness of two peer‐mentoring programs in improving employee retention rates in LTCS, among direct care staff	Pre and‐posttest, survey	Setting: 31 selected NHs (Growing Strong Roots) and 13 (Peer Mentoring for Long‐Term Care Charge Nurses) in New York, USA Participation rate: not reported Participant sample: not reported Response rate: not reported *Participant characteristics*: Gender: not reported Age: not reported Tenure: not reported Race: not reported	Retention rate: calculated by using start date of intervention group, the number of CNAs in the group, and the number of CNAs still employed by the facility 3 and 6 months after the intervention Retention rate at 3‐, 6‐ and 9‐month for Charge nurses – no further information provided	Descriptive analysis Paired‐samples *t*‐tests Arcsine transformation used to transform retention rate percentages Analysis of variances (ANOVA) and Lower‐bound test used to adjust for sphericity following Mauchly's test Least Significant Difference comparisons to compare retention rates among different groups and at different time points, evaluating the impact of peer‐mentoring interventions on staff retention in LTCFs	**Growing strong roots (CNAs)** *Study 1* Employee retention: significant for intervention group – baseline (51%) to 3‐month posttest (70%) (*t*‐value 4.08, *df* = 15, *p* < .001) represented a 19‐point increase or a 37% increase in retention rate *Study 2* Group 1's 3‐month postretention rate was significantly higher than Group 2's 6‐month retention rate (*p* < .02) Group 2's 3‐month retention rate was significantly higher than its 6‐month retention rate (*p* < .001) Group 3's data were eliminated due to insufficient data collection The baseline data were significantly different from each other (*p* < .01) **Peer mentoring for long‐term care charge nurses** At the 3‐month posttest mark of the peer‐mentoring program, retention rate increased from 75% at baseline to 91% for new charge nurses (*t* = 2.16, *df* = 12, *p* < .05)	2	No	Growing Strong Roots (CNAs) Potential bias as the study was conducted only in NHs which volunteered, limited generalisability due to data collected from a specific state, and these facilities had higher retention rates than general Not reported by Hegeman et al. for Study 2 Peer Mentoring for Long‐Term Care Charge Nurses Not reported by Hegeman et al.
Hunt et al. (2012), USA	To explore the relationships between retention strategies, employee benefits, features of the practice environment, and RN retention	Secondary analysis of cross‐sectional, survey	Setting: 1174 NHs selected across the USA, using NH characteristics to inform a stratified, multistage probability design Participation rate: 78% (1174/1500) Participant sample: not reported (14% RNs, 21% LPNs, 67% CNAs reported working in week prior) Response rate: not reported *Participant characteristics*: Gender: not reported Age: not reported Tenure: not reported Race: not reported	RN retention: percentage of RNs employed for more than 1 year categorised into: low (50% or less of RNs retained for more than 1 year), moderate (51%–79%), and high (80% or more) Workplace characteristics and Human Resource policies: categorised as intrinsic (recognition programs, paid conference attendance, career ladders, tuition reimbursement, career development programs, and attendance awards), coded 0/1; and extrinsic (paid personal days, retirement benefits, paid sick days, and medical insurance benefits), coded 0/1 Total hours per resident day, proxy for staffing adequacy Secondary data – 2004 National Nursing Home Survey	Descriptive statistics Analysis of variance *t*‐tests Weighted multinomial logistic regression with an incremental approach to analyse intrinsic, extrinsic and extrinsic‐control factors and RN retention	In Model 3 (high vs. moderate), longer DoN tenure (1.004, 95% CI, 1.001, 1.008) was positively associated with higher retention Offering attendance awards (.683, 95% CI, .495, .94) and the presence of an Alzheimer's unit (.667, 95% CI, .471, .943) was associated with lower retention levels in nursing homes	4	No	Findings not applicable to NHs without Medicare/Medicaid certification or licenced by state agencies, cross‐sectional design limits inferences of causality, administrator self‐report of RN retention, subjective selection of three RN retention groups, potential ambiguity in variable definitions, and exclusion of factors with missing values
Karmacharya (2023), USA	To identify perspectives of personnel in leadership and direct care on the work culture that promote retention in high‐performing NHs, AL and HCAs	Qualitative, using semi‐structured interviews	Setting: 12 purposively selected high‐performing NHs, ALs, and HCAs across Ohio, America. Participation rate: NH 50% (2/4) and then 67% (2/3); AL 100% (4/4); HCAs 75% (3/4) and then 100% (1/1) (4 NHs, 4 ALs, 4 HCAs) Participant sample: *n* = 37 21 LP, 16 DCWs **Participant characteristics**: *LP* Gender: not reported. Age: not reported. Tenure (average years): 10 (range 2.5–23 years.) Race: not reported *DCW* Gender: 100% female. Age: not reported. Tenure (average years): 7 with their organisation and 15 in direct care workforce (range 1–32 years). Race: not reported	Not reported	Thematic analysis	Three themes were associated with DCW retention: (1) family‐like organisational approach, (2) supportive working conditions, and (3) worker empowerment	5	No	This study did not capture racial identity and all DCWs identified as female. The findings may not reflect non‐female identifying DCWs' experiences. Retention strategies used from the beginning of the COVID‐19 pandemic were not included in data used to identify high‐performing NHs and ALs (collected in 2019)
Kennedy et al. (2020), USA	To examine the factors associated with turnover and retention rates of CNAs within NHs in Ohio	Secondary analysis of cross‐section, survey	Setting: 536 NHs (non‐hospital‐based) in Ohio, USA Participation rate: 64% (536/835) Participant sample: not reported Response rate: not reported *Participant characteristics*: Gender: not reported Age: not reported Tenure: not reported Race: not reported	Annual retention rate: CNAs employed in both first and last payroll of 2015 Covariates included facility structure, financial resources, staffing and management resident case mix, and county‐level job market conditions Facility attributes considered ownership type, number of beds, presence of dementia care units, and rural location Financial resources included occupancy rate and payer mix, while staffing and management variables comprised staffing levels, wages, turnover, consistent assignment, and staff empowerment Secondary data: 2017 Ohio Biennial Survey of LTCFs, Ohio Medicaid Cost Reports, Certification and Survey Provider Enhanced Reports, the Ohio Biennial Survey of Long‐Term Care Facilities, and the Area Health Resource File	Descriptive statistics Pearson correlation to assess the bivariate association between continuous measures of CNA retention and turnover rates *χ* ^2^ test to assess the bivariate relationship between the quartile categories of turnover and retention Ordinary least squares linear regression models to investigate whether the predictors of retention and turnover rates differed or not	Not‐for‐profit NHs (Coef. 3.30, SE 1.64, *p* < .05) were significantly associated with higher CNA retention	5	Yes	Self‐reported data without external verification, some significant differences in variables between the analytical sample and facilities excluded from analysis limiting generalisability, lack of information on additional factors influencing staff stability, the study being limited to one state (Ohio), and potential limitations in measuring CNA empowerment at the organisational level
Meyer et al. (2014), USA	To follow rural CNAs in the United States for 1‐year posttraining to identify retention and turnover issues in the LTC setting by exploring the CNAs perceptions of the LTC work experience	Longitudinal, survey	Setting: LTCFs purposively selected in rural area of Midwestern region of USA Participation rate: not reported Participant sample: 408 CNAs (128/408) *Response rate*: Baseline *n* = 408 6 months = 191 (67%) 12 months = 128 (31%) *Participant characteristics*: Gender: 90% female Age (years): 45% – <30, 31%, 30–44, 24% – 45–64, 0% – >65 Tenure: not reported Race: not reported	Three surveys: initial survey gathering demographic characteristics, CNAs currently working comprising of four sections (personal/employment demographics, reasons for working, training/preparedness, workplace issues) and not working in a LTCF also comprising four sections (work history, personal/employment demographics, workplace issues, comparison of current employment to working as CNA, factors influencing return to work) using 5‐point Likert‐type scale Surveys were conducted at the end of CNA training, and at 6‐ and 12‐month posttraining	Descriptive statistics Inferential statistics such as correlation, *χ* ^2^ and *t*‐tests were used as needed	No statistically significant differences found in retention related to demographic characteristics or the type of CNA training program attended (facility or non‐facility‐based training site). Additionally, there were no significant differences in training satisfaction between facility‐ and non‐facility‐based training sites	5	No	Small sample size due to high participant attrition over 12 = months, challenges in maintaining contact with a highly mobile population like CNAs, potential limitations in generalisability due to the rural setting of the study. Additionally, the initial contact point during CNA training may have limited the applicability of findings to CNAs with work experience
Mountford (2013), Australia	To investigate the application of human resource strategies aimed at the retention of DCWs in ACFs, specifically focusing on older workers	Qualitative, using semi‐structured interviews	Setting: 20 randomly selected ACFs across Sydney, Australia Facility managers nominated personal carers for interviews Participation rate: not reported Participant sample: *n* = 40 20 PCs, 20 FMs *Participant characteristics*: Gender: 18 female, 2 male Age: 100% 45 + years Tenure: not reported Race: 11 from NESB, 9 Australian born	Unreported by Montford. Authors interpretation of outcome measure: the first half of the FMs' questions provided statistical data on their ACF and staff, and the second half focusing on retention policies, strategies and practices. The PCs' questions were primarily complementary to the FMs'	Data were grouped and analysed based on key themes and categories derived from literature on supportive work practices including, supportive work environment, recognition of skills and respect, training with career paths and opportunities to pass on knowledge	Effective retention of older workers involved supportive work practices, including fostering a positive work environment, recognising skills and respect, providing training for career advancement, and transferring knowledge to younger staff	5	No	Study was conducted over 2 months impacting sample size and data collection. Lack of representativeness regarding employment status and cultural background, limited industry knowledge before conducting interviews. Narrow focus on HR strategies for retaining older workers in ACFs. PCs were chosen by their managers, preventing generalisability to all PCs and sample differed from industry norms. No interview guide, questions provided, length of interview time not reported, and very limited description of analysis making replication of study difficult and reduced confidence in rigour of methods
Pillemer et al. (2008), USA	To evaluate the effectiveness of a retention specialist model in reducing employee turnover in NH facilities	Pretest‐posttest, questionnaire	Setting: 32 stratified randomly selected NHs in New York (*n* = 24) and Connecticut (*n* = 8), USA Participation rate: not reported Participant sample: 762 CNAs who completed two or more assessments Response rate: 94% (30/32 facilities) **Participant characteristics**: *Treatment group* Gender: 93% female Age (years): 21% – 18–30, 23% – 31–40, 32% – 41–50 = 25% – >51 Tenure: not reported. Race: 45% white, 43% black *Control group* Gender: 92% female Age (years): 18% – 18–30, 22% – 31–40, 34% – 41–50 = 27% – >51 Tenure: not reported Race: 45% white, 47% black	Perceived facility retention effort: overall quality of retention efforts was measured (1) rating overall quality of retention efforts on a scale of 0–10 and (2) answer four questions using scale of 1 to 4 on the facilities retention efforts Attitudes toward the facility: dimensions of the quality of the facility where they worked were rated on a scale of 0–10 Job satisfaction: 11 item Generic Job Satisfaction Scale (MacDonald and MacIntyre 1997), using a scale of 1–4 Stress: single question, using a scale of 1–4	Descriptive statistics A 2 × 3 repeated measures design (Treatment × Time) formed the core of the statistical models for evaluation of the intervention. Statistical models included factors such as state, the facility size and setting, CNA characteristics and profit status, in addition to time and treatment, in the analysis General linear mixed model methods and their extensions to analyse the models The primary examination of the intervention's effectiveness was reported at three assessment times (baseline, 6‐ and 12‐month)	The *Rating of Quality Efforts to Retain Good Employees* in the Treatment group showed significant positive changes, from baseline to 6‐month: mean value of .876, *p* < .001 A potential drop‐off effect was observed between 6 and 12 months, (mean −.720, *p* < .001). The initial significant gain decreased, was not significant between Time 1 and Time 3, but the overall effect remained significant when averaged across both time points (baseline compared to 6‐ and 12‐month/2 mean .516, *p* < .002). For the Treatment Effect group significant positive effects were seen from baseline to 6‐month mean (*p* .001) and from baseline compared to 6‐ and 12‐month/2 mean (*p* < .009) The *Positive Retention Efforts Scale* in the Treatment group showed significant positive changes, from baseline to 6‐month: mean value of .488, *p* < .004 and a negative association from 6‐ to 12‐month: mean value of −.759, *p* < .001	1	No	Drop‐off in effects over time. Sustainability concerns arose as some positive effects were not maintained, raising questions about the retention specialist model's lasting impact on staff outcomes, and the impact of RS turnover and maintaining level of effort. Disparities in resources between larger and smaller facilities may have influenced program effectiveness, emphasising the importance of addressing resource discrepancies for intervention success
Rantz et al. (2010), USA	To test the unique and combined contributions of Electronic Medical Record at the bedside and on‐site consultation by gerontological expert nurses on cost, staffing and quality of care in NHs	Longitudinal, four group comparison (2 intervention and 2 control groups), organisational‐level data	Setting: 18 stratified purposively selected NHs in three states of USA Participation rate: not reported Participant sample: not reported Response rate: not reported *Participant characteristics*: Gender: not reported Age: not reported Tenure: not reported Race: not reported	Staff retention: data collected from each facility (staff dates of hire and job codes) to determine the impact of the intervention on staff turnover rates Resident outcomes: Minimum Data Set derived quality indicators and quality measures. Quality indicator scores calculated using standardised algorithms; downloaded publicly reported quality measure scores from public website Secondary data Costs and staffing: data from Medicaid cost reports	Descriptive analysis Relative change scores to compare changes in residents' outcomes across groups	The study did not identify any discernible trend in the staff retention over time in any of the four groups (*p* = .54)	3	No	Small convenience sample size of 18 NHs, potential self‐selection bias with participating facilities that were willing to adopt technology and associated costs, and a lack of detailed participant characteristics
Sabi Boun et al. (2023), Canada	To understand the confluence of factors that led CNAs to resign from their jobs during the first wave of the COVID‐19 crisis in Montreal's LTCFs	Qualitative, using semi‐structured interviews	Setting: 8 selected LTCs in Montreal, Canada Participation rate: not reported Participant sample: *n* = 11 11 CNAs *Participant characteristics*: Gender: 73% female Age (mean): 41 years Tenure: not reported Race: not reported	Interview guide based on two Canadian theoretical frameworks: nurses' retention and work‐family balance were developed	Thematic analysis	Two themes were associated with retention: (1) emotional attachment to the profession and patients, and (2) organisational and governmental support	5	No	Recruitment was difficult with participants declining or requesting compensation, potentially introducing bias. Social desirability bias was also a potential limitation
Salmond et al. (2017), USA	To determine the effects of the LTC Nurse Residency Program on new nurses' confidence, competence, retention, job satisfaction, and perceptions of organisational safety	Pretest‐posttest, mixed‐methods, survey, focus groups and interviews	Setting: 36 purposively selected LTCFs in New Jersey, USA **Quantitative** Participation rate: not reported Participant sample: *n* = 36 nurse residents Response rate: 97% (36/37) *Participant characteristics*: Nurse residents: Gender: 75% female Age (years): 36% – 21–29, 28% – 30–39, 22% – 40–49, 0% – >50 Tenure: not reported Race: 31% – Asian/Pacific Islander, 17% – Black, 25% – Caucasian, 6% – Hispanic, 3% – Other, 6% – Not wish to disclose, 14% – No response **Qualitative** Participation rate: not reported Participant sample: not reported Response rate: not reported *Participant characteristics*: NNR and FMs: Gender: not reported Age: not reported Tenure: not reported Race: not reported	**Quantitative** Staff perceptions of quality: NH survey on Patient Safety Culture, 44 items to measure 13 dimensions using 5‐point Likert scale Knowledge, attitudes, and perceptions of care for geriatric patients: Geriatric Institutional Assessment Profile used and data aggregated on staff knowledge, attitudes, and competence in caring for the older adult Moral and work satisfaction: Job Satisfaction Survey, seven dimensions, using 5‐point Likert scale Comfort, confidence and support: Casey‐Fink Graduate Nurse Experience Survey, using 4‐point Likert scale to measure 5 dimensions Perceptions of program: Modified Preceptorship Program Evaluation Tool: four dimensions, using 4‐point Likert scale Organisational level data were used to measure retention rates **Qualitative** No explicit mention of an interview guide, however question pertaining to retention asked during focus group session was – ‘does a new nurse residency program increase retention and job satisfaction in LTC environments?’	**Quantitative** Descriptive statistics Pretest‐posttest comparisons to assess changes in outcome measures before and after the nurse residency program **Qualitative** Not reported	**Quantitative** No statistically significant results relating to employee retention. The nurse residency program achieved an 86% retention rate among participants in the first year, in comparison to New Jersey's average RN retention rate of 54% **Qualitative** Nurse residents reported being more committed to the LTC due to the Program, whilst nurse residents reported on looking for other opportunities in acute care offering shift flexibility, increased pay and promotional/career opportunities. Heightened retention was also reported to be affected by a positive workplace environment	1	No	Small sample size of 36 participants, which may impact generalisability; challenges in data collection such as incomplete responses and difficulty accessing organisational data; and issues with survey completion due to participant constraints like time and forgotten passwords
Singh and Schwab (1998), USA	To measure the rate of turnover among NH administrators based on actual count of job changes within a 12‐month period and to investigate which specific dimensions in the administrator's job environment, along with administrator and organisational characteristics, influence retention	Cross‐sectional, survey	Setting: purposively selected NHs in Michigan and Indiana, USA Participation rate: not reported Participant sample: *n* = 552 NH administrators Response rate: 53% (552/1035) *Participant characteristics*: Gender: not reported Age: not reported Tenure (entire population): mean = 4.4 years Race: not reported	Retention: length of employment of 3 years or longer at the same facility Job‐related factors: 41 items (seven dimensions) about administrator's current job environment, using 4‐point scale Length of employment: mean, median, and mode were used to measure the length of employment among different subsets of administrators Realised Expectations: included 10 measures related to leadership style, ethical values, management philosophies, and goal achievement	Factor analysis Multiple regression analysis: job‐related factors and greater length of employment Pearson correlation: examined variables in three separate models (realised expectations, organisational demands and skill compatibility, commitment and retention)	In Model III, the retention of NH administrators was positively associated with facility size (Coef. .03, *p* ≤ .01), independent ownership (Coef. 2.96, *p* ≤ .05) and motivational commitment (Coef. 1.95, *p* ≤ .05)	4	Yes	Lack of a national sample and sample skewed towards not‐for‐profit facilities. Caution is needed in interpreting results due of ownership mix due to lack of data, doubts about generalisability despite meeting response rate criteria, and potential limitations in representing the diversity of ownership structures and facility types

*Note*: Sample size = the respective studies sample for analysis.

Abbreviations: ACF, Aged care facilities; AL, Assisted living; CNA, Certified nursing assistant; CW, Care worker; DCW, Direct care worker; DoN, Director of nursing; FM, Facility manager; FTE, Full‐time equivalent; HCA, Home care agencies; HR, Human resources; ITS, intention to stay; LP, Leadership personnel; LPN, Licensed practical nurse; LTC, Long‐term care; LTCF, Long‐term care facility; *N*, number; NA, Nursing assistant; NESB, Non‐English‐speaking background; NH, Nursing home; NHA, Nursing home administrator; NNR, New nurse resident; PC, Personal carer; RN, Registered nurse; SD, Standard deviation; T1, Time 1; T2, Time 2; USA, United States of America.

^a^ Variables demonstrating statistical significance were extracted and reported from the highest level of analysis.

**TABLE 3:** Factors and strategies promoting retention of aged care employees: Themes and sub‐themes.

Themes	Sub‐themes
1. Employee characteristics, stability and well‐being	Socio‐demographic characteristics Employee commitment and stability Employee well‐being and resilience
2. Workplace relationships	Leadership support Collaborative and supportive workplace relationships
3. Training opportunities and career growth	Skill development and professional growth Specialist training programs
4. Recognition, incentive and compensation	Employee recognition and appreciation Financial benefits
5. Organisational structure, culture and resources	Facility structure and contextual dynamics Organisational resources and specialist roles Adequate staffing levels Leadership continuity
6. Values driven and empowered care practice	Commitment to person‐centred care Employee empowerment and role flexibility

*Note*: The qualitative data presented in the table has been thematically analysed.

## 3 RESULTS

### 3.1 Search results

**FIGURE 1:**
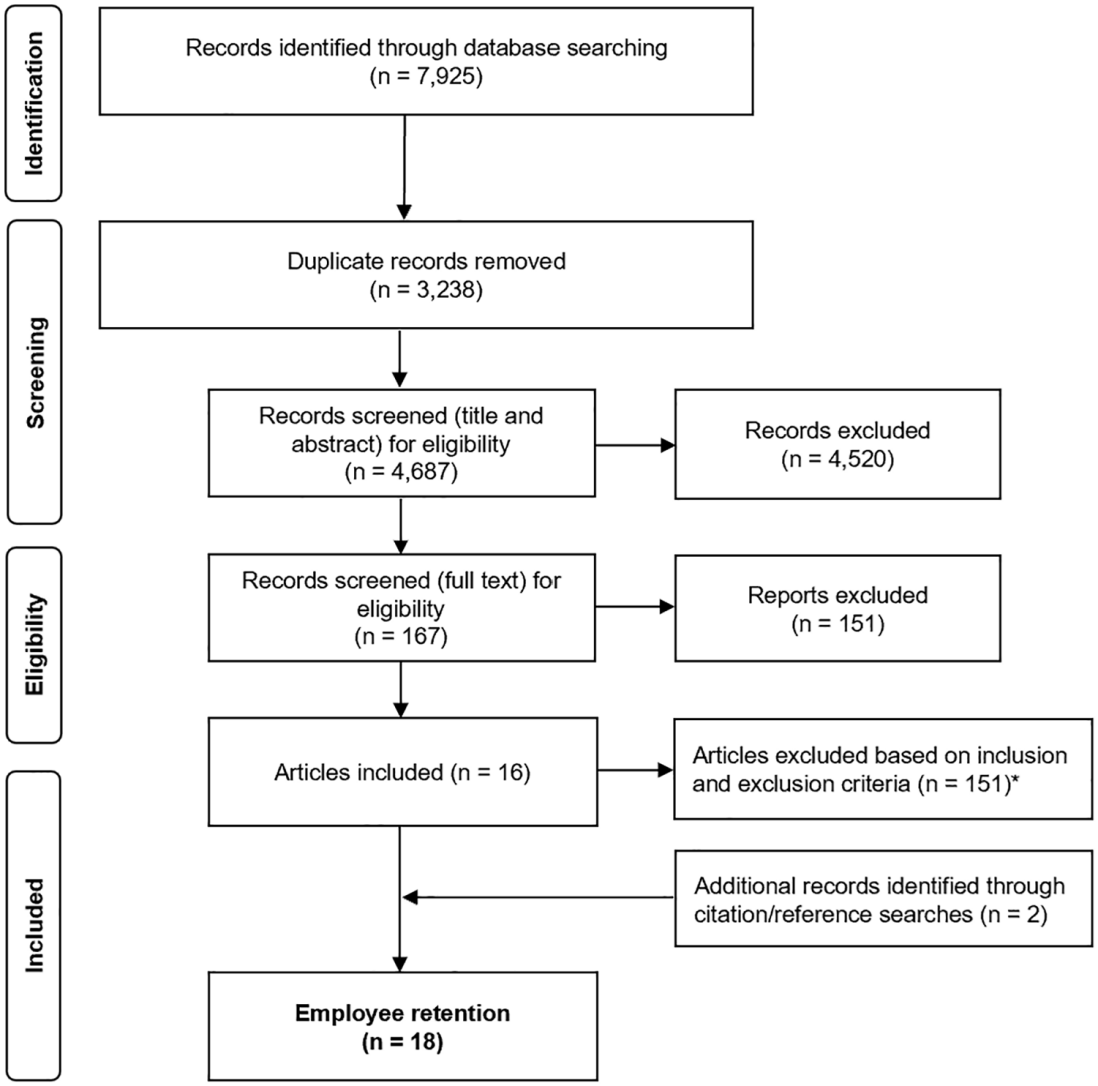
PRISMA flow diagram. ITL, Intention to leave; ITS, Intention to stay. *Articles excluded, with reasons (*n* = 151), including: Not retention support – studies that did not investigate the phenomenon of interest (*n* = 53), Not research – non‐primary research articles, rather than empirical studies (*n* = 37), Research findings – did not report of any impact pertaining to employee retention (*n* = 26), Focus Intention to stay, intention to leave or Turnover (*n* = 16), Population – studies where data were not collected from personal care workers' (*n* = 8), Setting – studies conducted in home care, community care or hospitals (*n* = 5), Differentiate results between recruitment and retention – studies that did not disaggregate findings based on recruitment and retention (*n* = 2), Abstract only: studies availability only in abstract form with a full‐text report (*n* = 2), Not research: non‐primary research articles, rather than empirical studies (*n* = 2).

The search yielded 7925 articles. After the removal of duplicates (*n* = 3238), the title and abstract screening resulted in 4520 articles being excluded. Full‐text screening excluded 151 articles, resulting in 16 included articles. Searching the reference lists of the 16 articles resulted in the inclusion of two additional articles (Figure 1).

### 3.2 Study characteristics

A summary of the main characteristics of the included studies is presented in Table 2, with additional information provided in Appendix S3. Eighteen articles, published between 1998 and 2023, were included in this review. Participant sample sizes across studies ranged from 36 to 2034. Fifteen studies were conducted in the United States of America (USA) and one each in Australia, Canada and Taiwan. The research designs comprised 13 quantitative studies, four qualitative studies and one mixed‐methods study. Seventeen studies were conducted in nursing homes (NH), with one study involving participants from various aged care settings, including NHs, assisted living facilities and home care. Of the 18 articles, only three included a definition of retention (Appendix S4).

### 3.3 Methodological quality of included studies

The methodological quality of included studies was assessed using the MMAT.^17^ Overall quality of the studies varied, with seven studies assessed as meeting all five methodological criteria (Table 2). Qualitative studies included in the review consistently demonstrated strong methodological quality. In contrast, quantitative studies, particularly the randomised controlled and non‐randomised studies, exhibited weaknesses, with some scoring as low as 1. This indicated substantial shortcomings with respect to sampling strategy, appropriateness of the selected measures, risk of bias, statistical analysis method, representativeness of the target population, incompleteness of the outcome data, confounders not being accounted for in the design and analysis, and whether the intervention (or exposure occurred) was administered as intended. The MMAT results are detailed in Appendix S5.

### 3.4 Factors and strategies promoting retention of aged care employees

The factors and strategies that promote the retention of employees working in the aged care sector identified in the 18 included studies were themed into six areas and are detailed below. Table 3 summarises the key themes and sub‐themes emerging from the data.

#### 3.4.1 Employee characteristics, stability and well‐being

Several socio‐demographic factors were significantly associated with employee retention.^20^ Retention rates were higher among older PCWs (46 years and over) compared to their younger counterparts (45 years and under).^20^ Marital status was positively influenced by retention across all age groups, while higher educational attainment (college level or above) was negatively associated with retention, particularly among older PCWs and the ‘total’ population.^20^

Beyond socio‐demographic factors, several employment‐related factors also significantly played a critical role in employee commitment and stability.^20^, ^21^, ^22^ A strong motivational commitment to the organisation among NH administrators^22^ and a greater intention to stay across the total population of PCWs^20^ were positively associated with retention. Conversely, long job tenure, prior health experience and being the primary breadwinner were negatively correlated with PCW retention.^21^

Employee well‐being and resilience were also key determinants of retention.^20^ Higher levels of depersonalisation, characterised by emotional detachment from residents, were significantly associated with increased retention among older PCWs,^20^ while optimisation strategies aimed at skill enhancement and goal achievement were also significantly associated with higher retention rates among older PCWs and the total sample.^20^ Conversely, emotional exhaustion negatively impacted retention, particularly among older PCWs and the total population.^20^

#### 3.4.2 Workplace relationships

Leadership support was consistently associated with higher retention among PCWs in multiple qualitative studies.^23^, ^24^, ^25^ Managers who were actively engaged,^24^, ^25^ emotionally supportive^24^ and maintained a visible presence in the workplace were linked to improved workforce stability. Open managerial communication and engagement, providing for employee involvement and input, were also perceived as enhancing retention among PCWs.^23^

Collaborative and supportive workplace relationships also played a critical role in retention outcomes.^23^, ^24^, ^26^, ^27^ Both formal and informal workplace connections were highly valued, particularly among medical directors, and were associated with increased retention.^27^ Additionally, perceptions of workplace collaboration^23^, ^24^ and supportive peer relationships among PCWs^26^ were perceived as key factors promoting retention across one quantitative and two qualitative studies.

#### 3.4.3 Training opportunities and career growth

Skill development and professional growth initiatives, including the provision of specialist training programs, yielded mixed outcomes in relation to employee retention.^23^, ^24^, ^25^, ^28^, ^29^, ^30^, ^31^ In several qualitative studies, organisations that provided training opportunities,^23^, ^24^ supported skill development^25^ and career progression^25^ were linked to improved retention among PCWs. However, quantitative results were inconsistent.^28^, ^29^, ^30^, ^31^ While a peer‐mentoring program targeting PCWs, RNs and LPNs (licensed practical nurse) demonstrated a modest but significant positive impact on retention, the effect diminished over time.^28^ Other training initiatives, such as compassion fatigue awareness and self‐care training for PCWs^29^ and a nurse residency program for newly graduated RNs,^31^ did not yield significant improvements in retention. Additionally, whether training was facility‐based or non‐facility‐based had no measurable significant effect on retention outcomes.^30^

#### 3.4.4 Recognition, incentive and compensation

Employee recognition was positively associated with retention across multiple studies.^23^, ^24^, ^32^ Employee recognition and expressions of appreciation,^24^, ^25^ employee respect,^25^ along with valuing and rewarding employee^23^ were perceived as contributing to higher retention among PCWs in three qualitative studies. However, attendance awards were negatively associated with retention in NHs with moderate RN retention rates, compared to those in the high RN retention rates.^32^

Financial benefits also played a significant role in workforce stability in multiple quantitative studies.^12^, ^21^, ^27^ Access to employee benefits, such as health insurance, was positively associated with higher PCW retention.^21^ However, findings on salary and retention varied across various roles.^12^ Higher PCW starting salaries were positively associated with improved RN and LPN retention, while higher LPN starting salaries were negatively associated with LPN retention.^12^ Among medical directors, financial factors such as fee schedules and on‐call stipends contributed to improved retention.^27^

#### 3.4.5 Organisational structure, culture and resources

Organisation structure and local market employment conditions significantly influenced retention outcomes.^2^, ^12^, ^22^, ^33^ Not‐for‐profit ownership was positively associated with higher retention rates,^2^ while for‐profit NHs were negatively associated with lower retention among PCWs.^12^, ^33^ Independently owned (stand‐alone) facilities and larger facility size were positively associated with increased retention among NH administrators.^22^ Additionally, higher occupancy rates were positively associated with increased PCW retention^12^, ^33^ and improved retention among RNs and LPNs,^12^ while higher local unemployment rates were associated with greater PCW retention,^12^ suggesting labour market influences workforce retention. Additionally, in a qualitative study, supportive working conditions, such as improved communication, support for worker morale, challenges of low‐paying jobs, and a family and home‐like environment were perceived to influence staff retention among PCWs.^25^

Workplace resources and amenities also played a role in staff retention^32^, ^34^, ^35^ Nursing homes with specialised Alzheimer's units were significantly associated with lower retention rates, particularly in NHs with moderate RN retention rates.^32^ While the introduction of a dedicated retention specialist initially significantly improved PCW retention, the effect declined after 6 and 12 months.^34^ Conversely, implementation of bedside electronic medical records system had no significant measurable effect on retention.^35^

Staff levels were also linked to retention outcomes in multiple quantitative studies.^12^, ^27^, ^33^ Longer work hours for RNs were positively associated with RN and PCW retention,^12^ while increased PCW, RN and LPN hours per resident per day contributed significantly to higher PCW retention.^33^ Conversely, LPN hours per resident per day were significantly negatively associated with reduced PCW retention, while increased LPN overtime was also associated with lower retention among PCW and RNs.^12^ Additionally, increased nursing staff, increased availability and role of registered staff, and on‐call coverage for medical practitioners contributed to increased retention among medical directors.^27^

Leadership continuity emerged as a key factor significantly influencing employee retention.^32^, ^33^, ^36^ Retention of personal care workers was significantly higher in NHs that reported one or two administrators in the past year, compared to those with three or more administrators during the same period.^33^ Additionally, a longer tenure of the director of nursing was positively associated with higher staff retention,^12^, ^32^ underscoring the role of stable leadership in fostering workforce commitment.

#### 3.4.6 Values driven and empowered care practice

Commitment to resident‐centred care was a key driver of retention.^23^, ^26^ In qualitative studies, emotional commitment and moral obligation to caring,^26^ along with a fulfilment in meeting residents' care needs^23^ were perceived to influence higher retention rates among PCWs.^26^

Organisational strategies emphasising employee empowerment and role flexibility were associated with improved retention among PCWs in four studies.^20^, ^23^, ^24^, ^25^, ^33^ Empowering employees through enabling authority, resources and decision‐making autonomy was positively and statistically significantly associated with higher retention among PCWs.^33^ An empowering work environment for PCWs was perceived to improve retention in a qualitative study,^24^ while a greater level of work latitude among older PCWs was positively and statistically significantly associated with a higher retention rate in a quantitative study.^20^ Job modifications, including job design and workforce adaptability to accommodate diverse employee needs and ages^23^ along with role flexibility,^25^ were perceived to promote retention among PCWs in two qualitative studies.

## 4 DISCUSSION

The primary objective of this integrative review was to synthesise the evidence on factors and strategies that promote employee retention in the aged care sector, revealing six key themes: (1) employee characteristics, stability and well‐being, (2) workplace relationships, (3) training opportunities and career growth, (4) recognition, incentive and compensation, (5) organisational structure, culture and resources and (6) values driven and empowered care practice. Most studies (15/18) were conducted in the United States, and the majority of studies focused on PCWs and were conducted in NH settings, with few addressing other aged care occupational groups, such as nurses, leadership personnel, and medical directors.

### 4.1 Quantitative and qualitative findings relating to employee retention in aged care

This review included 13 quantitative, four qualitative and one mixed‐methods study investigating factors and strategies to promote employee retention in the aged care sector. Application of the MMAT revealed notable methodological weaknesses, particularly among the quantitative studies. Greater confidence was placed in three of 10 quantitative descriptive studies and the four qualitative studies that met all five quality criteria, demonstrating strong coherence between the data collection, analysis and interpretation. In contrast, the greatest methodological limitations were exhibited in the randomised controlled and non‐randomised quantitative studies.

Quantitative studies in this review primarily identified relationships between socio‐demographic, employee stability, organisational and structural factors with employee retention, highlighting measurable predictors such as age, marital status, financial incentives, employee well‐being, staffing levels, leadership continuity and organisational structure. In contrast, the qualitative studies included in this review provided a deeper insight into the lived experience of employees, emphasising subjective factors such as employee recognition and appreciation, leadership support and intrinsic motivation. While the mixed‐methods study revealed no significant association between a nurse residency program and retention, an increased commitment to the organisation was linked to the program.

The findings from both quantitative and qualitative studies underscored the importance of job design, employee empowerment, supportive workplace relationships and opportunities for training and career development. However, discrepancies emerged in two intervention studies investigating the long‐term effectiveness of a specialised retention role and a training program, as well as in two descriptive studies exploring the impact of compensation for RNs, LPNs and medical directors on employee retention. In the two intervention studies, a randomised controlled trial investigating the effectiveness of a retention specialist^34^ and a non‐randomised trial investigating the effectiveness of a peer‐mentoring program,^28^ positive effects on retention outcomes were initially found. However, these effects diminished by the 6‐ and 12‐month follow‐ups.^28^, ^34^ Assessment with MMAT revealed low methodological quality for both studies. The randomised controlled trial scored 1/5 based on issues with sampling and statistical analysis, raising concerns about biases and the reliability and validity of the findings. The non‐randomised study scored 2/5 based on average quality in sample representativeness, confounder controls and the intervention duration. Of the 10 quantitative descriptive studies, two were assessed as good quality, scoring 3/5.

Second, findings relating to compensation and retention varied across staff roles in two descriptive studies.^12^, ^27^ In one study (scoring 5/5 demonstrating excellent quality), higher PCW starting salaries were positively associated with improved RN and LPN retention, while higher LPN starting salaries were negatively associated with LPN retention.^12^ In contrast, the other descriptive study (scoring 3/5 indicating good quality) showed that increased fee schedules and on‐call stipends contributed to improved retention among medical directors,^27^ though MMAT assessment revealed concerns regarding sample representativeness and potential non‐response bias.

The amalgamation of the quantitative and qualitative findings in this review highlights the need for a balanced approach to employee retention in aged care. While structural improvements are critical, they must be integrated with a range of employee, employer and organisation‐related factors and strategies, reinforcing the need for evidence‐informed initiatives to promote employee retention. Engaging the aged care workforce in co‐designing these strategies may additionally improve the effectiveness and sustainability of such initiatives.

### 4.2 Generalisability of the findings to the Australian context

Of the 13 studies included in this review, only one was conducted in Australia,^23^ while the majority of the studies were conducted in the United States. The generalisability of the findings may be influenced by structural and policy differences between the two countries, including variations in funding models, regulatory frameworks, employment conditions, workforce structures and care standards. However, the fundamental role, responsibilities and status of PCWs remain largely consistent across the two countries.^37^ In both Australia and the United States, PCWs spend a significant proportion of their time providing direct care and support to older people in residential aged care, often under challenging working conditions.^38^ These challenges include high workloads, staffing shortages, limited career progression opportunities, low wages, emotional and physical strain, and minimal professional recognition.^4^ The prevalence of these shared workforce issues suggests that while policy responses and interventions aimed at promoting employee retention must be tailored to Australia's specific regulatory and funding requirements, the findings of this review remain relevant to all aged care operators, policymakers, and researchers.

The findings of this review align with the recommendations of the two prominent reports arising from national inquiries into Australia's aged care workforce. These include the Aged Care Sector Workforce Strategy's *A Matter of Care* ^39^ and the Royal Commission into Aged Care Quality and Safety (RCACQS) (2018–2021) Final report titled *Care Dignity and Respect*,^4^ with the latter report driving Australia's aged care reforms.

We found that an employee's intrinsic motivation, particularly their focus on resident‐centred care, was linked to employee retention. This finding is echoed by both Australian reports, which highlight the relationship between intrinsic rewards of caring and the retention of aged care workers.^4^, ^39^ This suggests that fostering deeper emotional connections and a sense of purpose in caregiving roles may be essential to improving employee retention in aged care settings. This finding is particularly important with Australia recently passing a new Aged Care Bill 2024, reflecting a shift towards a rights‐based, person‐centred approach that prioritises the needs of older people,^40^ along with the revised strengthened Aged Care Quality Standards (2024), commencing on 1 July 2025, placing resident‐centred care at the forefront.^41^

Consistent with the findings of a recent systematic review by Thwaites et al.,^36^ this review revealed positive relationships and supportive leadership as key factors influencing employee retention in aged care. The RCACQ final report underscored the crucial role of leadership in workforce retention.^4^ Similarly, the Matter of Care report emphasised leadership as both a driver and enabler of engagement, fostering a stable workforce across various levels of staff.^39^ Consistent across these reports and reinforced by this review and published research on direct care worker retention,^42^, ^43^, ^44^ is recognition of the pivotal role of leadership in retaining a stable and competent aged care workforce. In strengthening leadership across Australia's aged care sector, several key reforms have been introduced. These include the revised Aged Care Quality Standards, particularly Standard 7: Organisational Governance, which emphasises greater leadership accountability, and at an operational level, the implementation of the 24/7 RN requirement in residential aged care, which mandates the minimum amount of time for RN coverage of direct care provided to residents, while providing clinical leadership and giving PCWs guidance and decision‐making support.^45^

Additionally, organisational factors were found in most included studies in this review to be key to employee retention, highlighting the significant role organisations play in retaining PCWs. Although the findings of this review reveal mixed results regarding the influence of organisational ownership on staff retention, historically, not‐for‐profit residential operators have played a critical role in Australia's aged care sector, offering a substantial portion of residential care services. In recent years, mounting financial pressures have driven consolidation and mergers, leading to growth among for‐profit operators.^46^ The combination of lower employee retention in for‐profit facilities, as revealed in this review,^12^ and the significant growth of the for‐profit sector could exacerbate workforce instability, potentially compromising care quality, as for‐profit operators continue to manage a significant portion of the market.

### 4.3 Definitional ambiguity in employee retention research

While retention is generally understood as the length of employees' tenure and indicates an organisation's ability to retain personnel over time,^12^ most authors did not provide a definition against which they were measuring retention. The absence of a universally accepted definition of ‘retention’ in the literature presents a challenge to benchmarking and comparing research findings. Of the 18 included articles, only three included a definition of retention,^2^, ^12^, ^22^ with each using a different definition (Appendix S4). In one study, retention was defined by average employment duration at the same facility.^12^ In another, retention was measured as a percentage of staff employed over a specific time.^2^ In the third, retention was defined as employment of 3 years or longer at the same facility.^22^ Establishing a clear, widely accepted and consistently applied definition of retention is essential to facilitate cross‐study synthesis, to accurately identify retention rates, and to further enable evidence‐informed retention factors and strategies.

### 4.4 Strengths and limitations

The strength of this study lies in the rigour and comprehensiveness of the review process, guided by the PRISMA framework^16^ and Whittemore and Knafl's integrative review methodology.^15^ The review search strategy included a wide range of databases and qualitative, quantitative and mixed‐methods research methodologies. Additionally, a key strength of this review was the focus on a single occupational group, PCWs. Globally, despite the variations in aged care systems, the role and responsibilities of PCWs remain consistent across the included studies, enhancing the generalisability of the findings in relation to employee retention beyond the countries that were examined. However, despite developing a comprehensive search strategy in consultation with a health sciences librarian, there remains a possibility that some studies were missed. The term ‘retention’ was broadly used in all articles in this review, with most authors (15/18) failing to define retention. Most studies in this review were conducted in residential care, potentially limiting the translation of findings to the home care setting. Further, most studies employed cross‐sectional designs, limiting the ability to establish causation between the preselected variables under examination.

## 5 CONCLUSIONS

This review captured methodological and statistical heterogeneity in research studies exploring retention factors and strategies employed by aged care operators to retain their staff. Employee retention is a complex issue influenced by multiple factors, and this review underscores that no single factor can solely account for workforce retention. The retention of a stable and competent aged care workforce depends on a multifaceted approach.

Workforce retention is widely recognised as essential for maintaining a stable and skilled workforce. This review identified key factors and strategies across employee, employer and organisational domains that were found to influence retention among PCW in NHs. The retention of a stable and competent aged care workforce hinges on a multifaceted approach, incorporating employee, employer and organisational factors and strategies. The findings align with both government reports driving the transformation of Australia's aged care sector and peer‐reviewed literature, which consistently identifies job satisfaction and personal fulfilment as critical strategies to maintaining a stable, competent aged care workforce. As Australia progresses with generational reforms to deliver high‐quality, rights‐based person‐centred care that prioritises the needs of older people, implementing the evidence‐informed retention factors and strategies identified as influential in this review is essential.

Despite the critical importance of retaining the aged care workforce, particularly direct care workers, this review identified a paucity of evidence, especially regarding home care workers. The findings from this review are recommended for use by aged care operators, policymakers and researchers as a critical first step to guide the development, implementation and evaluation of evidence‐informed retention factors and strategies. Given the unique challenges in workforce retention within aged care, these findings provide valuable insights into evidence‐informed factors and strategies that promote retention and highlight the need for further empirical studies, particularly among former personal care workers and home care workers, given the lack of evidence in this area. Additionally, the development of a universal definition of ‘retention’ is needed, as the term is often used without clarity or consistency.

## CONFLICT OF INTEREST STATEMENT

No conflicts of interest declared.

## Supporting information


Appendix S1

Appendix S2

Appendix S3

Appendix S4

Appendix S5

## Data Availability

Data may be available from the corresponding author upon request.
