# Supplementary material for: A systematic, integrative review exploring supports that promote the retention of employees working in the aged care sector
Source: Australas J Ageing. 2025 Jul 31;44(3):e70070. doi: 10.1111/ajag.70070 (PMC12312298; doi:10.1111/ajag.70070)
Supplement: Supplementary file 1 — Appendix S1 [file AJAG-44-0-s004.docx]

**Appendix 1**

**Table 1:** Search strategy

APA PsycInfo (EBSCO)

(Date limited 1997 – 24 June 2024)

| **No.** | **Query** |
| --- | --- |
| S64 | S16 AND S38 AND S49 AND S62 |
| S63 | S16 AND S38 AND S49 AND S62 |
| S62 | S50 OR S51 OR S52 OR S53 OR S54 OR S55 OR S56 OR S57 OR S58 OR S59 OR S60 OR S61 |
| S61 | TI “employe* incentive program*” OR AB “employe* inventive program*” |
| S60 | TI “employe* assistan* program*” OR AB “employe* assistan* program*” |
| S59 | TI recommendation* OR AB recommendation* |
| S58 | TI incentive* OR AB incentive* |
| S57 | TI principle* OR AB principle* |
| S56 | TI protocol* OR AB protocol* |
| S55 | TI framework* OR AB framework* |
| S54 | TI initiative* OR AB initiative* |
| S53 | TI intervention* OR AB intervention* |
| S52 | TI train* OR AB train* |
| S51 | TI program* OR AB program* |
| S50 | TI strateg* OR AB strateg* |
| S49 | S39 OR S40 OR S41 OR S42 OR S43 OR S44 OR S45 OR S46 OR S47 OR S48 |
| S48 | TI “community aged care” OR AB “community aged care” |
| S47 | TI “home health care” OR AB “home health care” |
| S46 | TI “care at home” OR AB “care at home” |
| S45 | TI “home care” OR AB “home care” |
| S44 | TI “assisted living facilit*” OR AB “assisted living facilit*” |
| S43 | TI “home* for the aged” OR AB “home* for the aged” |
| S42 | TI ("long term care" OR "long-term care") OR AB ("long term care" OR "long-term care") |
| S41 | TI “nurs* home*” OR AB “nurs* home*” |
| S40 | TI “aged care” OR AB “aged care” |
| S39 | TI residential OR AB residential |
| S38 | S17 OR S18 OR S19 OR S20 OR S21 OR S22 OR S23 OR S24 OR S25 OR S26 OR S27 OR S28 OR S29 OR S30 OR S31 OR S32 OR S33 OR S34 OR S35 OR S36 OR S37 |
| S37 | MM "Top Level Managers" |
| S36 | MM "Medical Personnel" |
| S35 | TI “aged care*” OR AB “aged care*” |
| S34 | TI nurs* OR AB nurs* |
| S33 | TI healthcare OR AB healthcare |
| S32 | TI health OR AB health |
| S31 | TI “non-clinical” OR AB “non-clinical” |
| S30 | TI clinical OR AB clinical |
| S29 | TI “front line” OR AB “front line” |
| S28 | TI frontline OR AB frontline |
| S27 | TI “direct care” OR AB “direct care” |
| S26 | TI directcare OR AB directcare |
| S25 | TI manage* OR AB manage* |
| S24 | TI executive* OR AB executive* |
| S23 | TI “team leader*” OR AB “team leader*” |
| S22 | TI supervisor* OR AB supervisor* |
| S21 | TI personnel* OR AB personnel* |
| S20 | TI employe* OR AB employe* |
| S19 | TI worker* OR AB worker* |
| S18 | TI staff* OR AB staff* |
| S17 | TI workforce* OR AB workforce* |
| S16 | S1 OR S2 OR S3 OR S4 OR S5 OR S6 OR S7 OR S8 OR S9 OR S10 OR S11 OR S12 OR S13 OR S14 OR S15 |
| S15 | MM "Behavioral Intention" |
| S14 | MM "Employee Turnover" |
| S13 | TI stay* OR AB stay* |
| S12 | TI quit* OR AB quit* |
| S11 | TI leave* OR AB leave* |
| S10 | TI attrition OR AB attrition |
| S9 | TI retention OR AB retention |
| S8 | TI shortage OR AB shortage |
| S7 | TI voluntary OR AB voluntary |
| S6 | TI “turnover* rate*” OR AB “turnover* rate*” |
| S5 | TI “actual turnover*” OR AB “actual turnover*” |
| S4 | TI turnover* OR AB turnover* |
| S3 | TI “intention* to stay” OR AB “intention* to stay” |
| S2 | TI “turnover intention*” OR AB “turnover intention*” |
| S1 | TI “intention* to leave” OR AB “intention* to leave” |

Business Source Complete (EBSCO)

(Date limited 1997 – 24 June 2024)

| **No.** | **Query** |
| --- | --- |
| S66 | S15 AND S39 AND S51 AND S64 |
| S65 | S15 AND S39 AND S51 AND S64 |
| S64 | S52 OR S53 OR S54 OR S55 OR S56 OR S57 OR S58 OR S59 OR S60 OR S61 OR S62 OR S63 |
| S63 | TI “employe* incentive program*” OR AB “employe* inventive program*” |
| S62 | TI “employe* assistan* program*” OR AB “employe* assistan* program*” |
| S61 | TI recommendation* OR AB recommendation* |
| S60 | TI incentive* OR AB incentive* |
| S59 | TI principle* OR AB principle* |
| S58 | TI protocol* OR AB protocol* |
| S57 | TI framework* OR AB framework* |
| S56 | TI initiative* OR AB initiative* |
| S55 | TI intervention* OR AB intervention* |
| S54 | TI train* OR AB train* |
| S53 | TI program* OR AB program* |
| S52 | TI strateg* OR AB strateg* |
| S51 | S40 OR S41 OR S42 OR S43 OR S44 OR S45 OR S46 OR S47 OR S48 OR S49 OR S50 |
| S50 | DE "HOME care services |
| S49 | TI “community aged care” OR AB “community aged care” |
| S48 | TI “home health care” OR AB “home health care” |
| S47 | TI “care at home” OR AB “care at home” |
| S46 | TI “home care” OR AB “home care” |
| S45 | TI “assisted living facilit*” OR AB “assisted living facilit*” |
| S44 | TI “home* for the aged” OR AB “home* for the aged” |
| S43 | TI ("long term care" OR "long-term care") OR AB ("long term care" OR "long-term care") |
| S42 | TI “nurs* home*” OR AB “nurs* home*” |
| S41 | TI “aged care” OR AB “aged care” |
| S40 | TI residential OR AB residential |
| S39 | S16 OR S17 OR S18 OR S19 OR S20 OR S21 OR S22 OR S23 OR S24 OR S25 OR S26 OR S27 OR S28 OR S29 OR S30 OR S31 OR S32 OR S33 OR S34 OR S35 OR S36 OR S37 OR S38 |
| S38 | DE "PHYSICIANS" |
| S37 | DE "HEALTH services administrators" |
| S36 | DE "HEALTH practitioners" |
| S35 | DE "MEDICAL personnel" |
| S34 | TI “aged care*” OR AB “aged care*” |
| S33 | TI nurs* OR AB nurs* |
| S32 | TI healthcare OR AB healthcare |
| S31 | TI health OR AB health |
| S30 | TI “non-clinical” OR AB “non-clinical |
| S29 | TI clinical OR AB clinical |
| S28 | TI frontline OR AB frontline |
| S27 | TI “front line” OR AB “front line” OR frontline |
| S26 | TI directcare OR AB directcare |
| S25 | TI “direct care” AB “direct care” |
| S24 | TI manage* OR AB manage* |
| S23 | TI executive* OR AB executive* |
| S22 | TI “team leader*” OR AB “team leader*” |
| S21 | TI supervisor* OR AB supervisor* |
| S20 | TI personnel* OR AB personnel* |
| S19 | TI employe* OR AB employe* |
| S18 | TI worker* OR AB worker* |
| S17 | TI staff* OR AB staff* |
| S16 | TI workforce* OR AB workforce* |
| S15 | S1 OR S2 OR S3 OR S4 OR S5 OR S6 OR S7 OR S8 OR S9 OR S10 OR S11 OR S12 OR S13 OR S14 |
| S14 | DE "RESIGNATION of employees" |
| S13 | DE "LABOR turnover" |
| S12 | TI quit* OR AB quit* |
| S11 | TI leave* OR AB leave* |
| S10 | TI attrition OR AB attrition |
| S9 | TI retention OR AB retention |
| S8 | TI shortage OR AB shortage |
| S7 | TI voluntary OR AB voluntary |
| S6 | TI “turnover* rate*” OR AB “turnover* rate*” |
| S5 | TI “actual turnover*” OR AB “actual turnover*” |
| S4 | TI turnover* OR AB turnover* |
| S3 | TI “intention* to stay” OR AB “intention* to stay” |
| S2 | TI “turnover intention*” OR AB “turnover intention*” |
| S1 | TI “intention* to leave” OR AB “intention* to leave” |

CINHAL Complete (EBSCO)

(Date limited 1997 – 24 June 2024)

| **No.** | **Query** |
| --- | --- |
| S60 | S14 AND S34 AND S45 AND S58 |
| S59 | S14 AND S34 AND S45 AND S58 |
| S58 | S46 OR S47 OR S48 OR S49 OR S50 OR S51 OR S52 OR S53 OR S54 OR S55 OR S56 OR S57 |
| S57 | TI “employe* incentive program*” OR AB “employe* inventive program*” |
| S56 | TI “employe* assistan* program*” OR AB “employe* assistan* program*” |
| S55 | TI recommendation* OR recommendation* |
| S54 | TI incentive* OR AB incentive* |
| S53 | TI principle* OR AB principle* |
| S52 | TI protocol* OR AB protocol* |
| S51 | TI framework* OR AB framework* |
| S50 | TI initiative* OR AB initiative* |
| S49 | TI intervention* OR AB intervention* |
| S48 | TI train* OR AB train* |
| S47 | TI program* OR AB program* |
| S46 | TI strateg* OR AB strateg* |
| S45 | S35 OR S36 OR S37 OR S38 OR S39 OR S40 OR S41 OR S42 OR S43 OR S44 |
| S44 | TI “community aged care” OR AB “community aged care” |
| S43 | TI “home health care” OR AB “home health care” |
| S42 | TI “care at home” OR AB “care at home” |
| S41 | TI “home care” OR AB “home care” |
| S40 | TI “assisted living facilit*” OR AB “assisted living facilit*” |
| S39 | TI “home* for the aged” OR AB “home* for the aged” |
| S38 | TI ("long term care" OR "long-term care") OR AB ("long term care" OR "long-term care") |
| S37 | TI “nurs* home*” OR AB “nurs* home*” |
| S36 | TI “aged care” OR AB “aged care” |
| S35 | TI residential OR AB residential |
| S34 | S15 OR S16 OR S17 OR S18 OR S19 OR S20 OR S21 OR S22 OR S23 OR S24 OR S25 OR S26 OR S27 OR S28 OR S29 OR S30 OR S31 OR S32 OR S33 |
| S33 | TI “aged care*” OR AB “aged care*” |
| S32 | TI nurs* OR AB nurs* |
| S31 | TI healthcare OR AB healthcare |
| S30 | TI health OR AB health |
| S29 | TI “non-clinical” OR AB “non-clinical” |
| S28 | TI clinical OR AB clinical |
| S27 | TI frontline OR AB frontline |
| S26 | TI “front line” OR AB “front line” |
| S25 | TI directcare OR AB directcare |
| S24 | TI “direct care” OR AB “direct care” |
| S23 | TI manage* OR AB manage* |
| S22 | TI executive* OR AB executive* |
| S21 | TI “team leader*” OR AB “team leader*” |
| S20 | TI supervisor* OR AB supervisor* |
| S19 | TI personnel* OR AB personnel* |
| S18 | TI employe* OR AB employe* |
| S17 | TI worker* OR AB worker* |
| S16 | TI staff* OR AB staff* |
| S15 | TI workforce* OR AB workforce* |
| S14 | S1 OR S2 OR S3 OR S4 OR S5 OR S6 OR S7 OR S8 OR S9 OR S10 OR S11 OR S12 OR S13 |
| S13 | TI stay* OR AB stay* |
| S12 | TI quit* OR AB quit* |
| S11 | TI leave* OR AB leave* |
| S10 | TI attrition OR AB attrition |
| S9 | TI retention OR AB retention |
| S8 | TI shortage OR AB shortage |
| S7 | TI voluntary OR AB voluntary |
| S6 | TI “turnover* rate*” OR AB “turnover* rate*” |
| S5 | TI “actual turnover*” OR AB “actual turnover*” |
| S4 | TI turnover* OR AB turnover* |
| S3 | TI “intention* to stay” OR AB “intention* to stay” |
| S2 | TI “turnover intention*” OR AB “turnover intention*” |
| S1 | TI “intention* to leave” OR AB “intention* to leave” |

Medline Complete (EBSCO)

(Date limited 1997 – 24 June 2024)

| **No.** | **Query** |
| --- | --- |
| S63 | S15 AND S36 AND S48 AND S61 |
| S62 | S15 AND S36 AND S48 AND S61 |
| S61 | S49 OR S50 OR S51 OR S52 OR S53 OR S54 OR S55 OR S56 OR S57 OR S58 OR S59 OR S60 |
| S60 | TI “employe* incentive program*” OR AB “employe* inventive program*” |
| S59 | TI “employe* assistan* program*” OR AB “employe* assistan* program*” |
| S58 | TI recommendation* OR AB recommendation* |
| S57 | TI incentive* OR AB incentive* |
| S56 | TI principle* OR AB principle* |
| S55 | TI protocol* OR AB protocol* |
| S54 | TI framework* OR AB framework* |
| S53 | TI initiative* OR AB initiative* |
| S52 | TI intervention* OR AB intervention* |
| S51 | TI train* OR AB train* |
| S50 | TI program* OR AB program* |
| S49 | TI strateg* OR AB strateg* |
| S48 | S37 OR S38 OR S39 OR S40 OR S41 OR S42 OR S43 OR S44 OR S45 OR S46 OR S47 |
| S47 | MM "Health Services for the Aged" |
| S46 | TI “community aged care” OR AB “community aged care” |
| S45 | TI “home health care” OR AB “home health care” |
| S44 | TI “care at home” OR AB “care at home” |
| S43 | TI “home care” OR AB “home care” |
| S42 | TI “assisted living facilit*” OR AB “assisted living facilit*” |
| S41 | TI “home* for the aged” OR AB “home* for the aged” |
| S40 | TI ("long term care" OR "long-term care") OR AB ("long term care" OR "long-term care") |
| S39 | TI “nurs* home*” OR AB “nurs* home*” |
| S38 | TI “aged care” OR AB “aged care” |
| S37 | TI residential OR AB residential |
| S36 | S16 OR S17 OR S18 OR S19 OR S20 OR S21 OR S22 OR S23 OR S24 OR S25 OR S26 OR S27 OR S28 OR S29 OR S30 OR S31 OR S32 OR S33 OR S34 OR S35 |
| S35 | MM "Health Personnel+" |
| S34 | TI “aged care*” OR AB “aged care*” |
| S33 | TI nurs* OR AB nurs* |
| S32 | TI healthcare OR AB healthcare |
| S31 | TI health OR AB health |
| S30 | TI “non-clinical” OR AB “non-clinical |
| S29 | TI clinical OR AB clinical |
| S28 | TI frontline OR AB frontline |
| S27 | TI “front line” OR AB “front line” |
| S26 | TI directcare OR AB directcare |
| S25 | TI “direct care” OR AB “direct care” |
| S24 | TI manage* OR AB manage* |
| S23 | TI executive* OR AB executive* |
| S22 | TI “team leader*” OR AB “team leader*” |
| S21 | TI supervisor* OR AB supervisor* |
| S20 | TI personnel* OR AB personnel* |
| S19 | TI employe* OR AB employe* |
| S18 | TI worker* OR AB worker* |
| S17 | TI staff* OR AB staff* |
| S16 | TI workforce* OR AB workforce* |
| S15 | S1 OR S2 OR S3 OR S4 OR S5 OR S6 OR S7 OR S8 OR S9 OR S10 OR S11 OR S12 OR S13 OR S14 |
| S14 | MM "Personnel Turnover" |
| S13 | TI stay* OR AB stay* |
| S12 | TI quit* OR AB quit* |
| S11 | TI leave* OR AB leave* |
| S10 | TI attrition OR AB attrition |
| S9 | TI retention OR AB retention |
| S8 | TI shortage OR AB shortage |
| S7 | TI voluntary OR AB voluntary |
| S6 | TI “turnover* rate*” OR AB “turnover* rate*” |
| S5 | TI “actual turnover*” OR AB “actual turnover*” |
| S4 | TI turnover* OR AB turnover* |
| S3 | TI “intention* to stay” OR AB “intention* to stay” |
| S2 | TI “turnover intention*” OR AB “turnover intention*” |
| S1 | TI “intention* to leave” OR AB “intention* to leave” |

Embase (Embase.com)

(Date limited 1997 – 24 June 2024)

| No. | Query |
| --- | --- |
| #60 | #58 AND 'article'/it AND [english]/lim |
| #59 | #58 AND 'Article'/it |
| #58 | #14 AND #32 AND #44 AND #57 |
| #57 | #45 OR #46 OR #47 OR #48 OR #49 OR #50 OR #51 OR #52 OR #53 OR #54 OR #55 OR #56 |
| #56 | 'employe* incentive program*':ab,ti AND [embase]/lim AND [1997-2022]/py |
| #55 | 'employe* assistan* program*':ab,ti AND [embase]/lim AND [1997-2022]/py |
| #54 | recommendation*:ab,ti AND [embase]/lim AND [1997-2022]/py |
| #53 | incentive*:ab,ti AND [embase]/lim AND [1997-2022]/py |
| #52 | principle*:ab,ti AND [embase]/lim AND [1997-2022]/py |
| #51 | protocol*:ab,ti AND [embase]/lim AND [1997-2022]/py |
| #50 | framework*:ab,ti AND [embase]/lim AND [1997-2022]/py |
| #49 | initiative*:ab,ti AND [embase]/lim AND [1997-2022]/py |
| #48 | intervention*:ab,ti AND [embase]/lim AND [1997-2022]/py |
| #47 | train*:ab,ti AND [embase]/lim AND [1997-2022]/py |
| #46 | program*:ab,ti AND [embase]/lim AND [1997-2022]/py |
| #45 | strateg*:ab,ti AND [embase]/lim AND [1997-2022]/py |
| #44 | #33 OR #34 OR #35 OR #36 OR #37 OR #38 OR #39 OR #40 OR #41 OR #42 OR #43 |
| #43 | 'aged care':ab,ti AND [embase]/lim AND [1997-2022]/py |
| #42 | 'community aged care':ab,ti AND [embase]/lim AND [1997-2022]/py |
| #41 | 'home health care':ab,ti AND [embase]/lim AND [1997-2022]/py |
| #40 | 'care at home':ab,ti AND [embase]/lim AND [1997-2022]/py |
| #39 | 'home care':ab,ti AND [embase]/lim AND [1997-2022]/py |
| #38 | 'assisted living facilit*':ab,ti AND [embase]/lim AND [1997-2022]/py |
| #37 | 'home* for the aged':ab,ti AND [embase]/lim AND [1997-2022]/py |
| #36 | 'long-term care':ab,ti AND [embase]/lim AND [1997-2022]/py |
| #35 | 'long term care':ab,ti AND [embase]/lim AND [1997-2022]/py |
| #34 | 'nurs* home*':ab,ti AND [embase]/lim AND [1997-2022]/py |
| #33 | residential:ab,ti AND [embase]/lim AND [1997-2022]/py |
| #32 | #15 OR #16 OR #17 OR #18 OR #19 OR #20 OR #21 OR #22 OR #23 OR #24 OR #25 OR #26 OR #27 OR #28 OR #29 OR #30 OR #31 |
| #31 | 'aged care':ab,ti AND [embase]/lim AND [1997-2022]/py |
| #30 | nurs*:ab,ti AND [embase]/lim AND [1997-2022]/py |
| #29 | healthcare:ab,ti AND [embase]/lim AND [1997-2022]/py |
| #28 | health:ab,ti AND [embase]/lim AND [1997-2022]/py |
| #27 | 'non-clinical':ab,ti AND [embase]/lim AND [1997-2022]/py |
| #26 | clinical:ab,ti AND [embase]/lim AND [1997-2022]/py |
| #25 | 'front line':ab,ti AND [embase]/lim AND [1997-2022]/py |
| #24 | 'direct care':ab,ti AND [embase]/lim AND [1997-2022]/py |
| #23 | manage*:ab,ti AND [embase]/lim AND [1997-2022]/py |
| #22 | executive*:ab,ti AND [embase]/lim AND [1997-2022]/py |
| #21 | 'team leader*':ab,ti AND [embase]/lim AND [1997-2022]/py |
| #20 | supervisor*:ab,ti AND [embase]/lim AND [1997-2022]/py |
| #19 | personnel*:ab,ti AND [embase]/lim AND [1997-2022]/py |
| #18 | employe*:ab,ti AND [embase]/lim AND [1997-2022]/py |
| #17 | worker*:ab,ti AND [embase]/lim AND [1997-2022]/py |
| #16 | staff:ab,ti AND [embase]/lim AND [1997-2022]/py |
| #15 | workforce:ab,ti AND [embase]/lim AND [1997-2022]/py |
| #14 | #1 OR #2 OR #3 OR #4 OR #5 OR #6 OR #7 OR #8 OR #9 OR #10 OR #11 OR #12 OR #13 |
| #13 | stay:ab,ti AND [embase]/lim AND [1997-2022]/py |
| #12 | quit:ab,ti AND [embase]/lim AND [1997-2022]/py |
| #11 | leave:ab,ti AND [embase]/lim AND [1997-2022]/py |
| #10 | attrition:ab,ti AND [embase]/lim AND [1997-2022]/py |
| #9 | retention:ab,ti AND [embase]/lim AND [1997-2022]/py |
| #8 | shortage:ab,ti AND [embase]/lim AND [1997-2022]/py |
| #7 | voluntary:ab,ti AND [embase]/lim AND [1997-2022]/py |
| #6 | 'turnover rate*':ab,ti AND [embase]/lim AND [1997-2022]/py |
| #5 | 'actual turnover*':ab,ti AND [embase]/lim AND [1997-2022]/py |
| #4 | turnover*:ab,ti AND [embase]/lim AND [1997-2022]/py |
| #3 | 'intention* to stay':ab,ti AND [embase]/lim AND [1997-2022]/py |
| #2 | 'turnover intention*':ab,ti AND [embase]/lim AND [1997-2022]/py |
| #1 | 'intention* to leave':ab,ti AND [embase]/lim AND [1997-2022]/py |
