# Supplementary material for: A systematic, integrative review exploring supports that promote the retention of employees working in the aged care sector
Source: Australas J Ageing. 2025 Jul 31;44(3):e70070. doi: 10.1111/ajag.70070 (PMC12312298; doi:10.1111/ajag.70070)
Supplement: Supplementary file 2 — Appendix S2 [file AJAG-44-0-s001.docx]

**Appendix 2**

**Table 1:** Combined quantitative and qualitative findings

| **Themes** | **Sub-themes** |
| --- | --- |
| **1. Employee characteristics, wellbeing and stability** | Socio-demographic characteristics  Employee commitment and stability  Employee well-being and resilience |
| **2. Workplace relationships** | Leadership support  Collaborative and supportive workplace relationships |
| **3. Training and professional development** | Specialist training programs  Skill development and professional growth |
| **4. Recognition, incentive and compensation** | Employee recognition and appreciation  Financial benefits |
| **5. Organisational structure and resources** | Facility structure and contextual dynamics  Organisational resources and specialist roles  Adequate staffing levels  Leadership continuity |
| **6. Values driven and empowered care practice** | Commitment to person-centred care  Employee empowerment and role flexibility |

**Note:** The qualitative data presented in the table, has been thematically analysed

1. **Employee demographics and well-being**

**Socio-demographic characteristics**

- - Gender (younger workers)^20^
  - Educational attainment (older workers/total population)^20^
  - Marital status (younger/older/both younger and older workers) ^20^

**Employee commitment and stability**

- - Breadwinner – primary household earner^21^
  - Past health experience^21^
  - Tenure in job^21^
  - Intention to stay (total population) ^20^
  - Motivational commitment^22^

**Employee well-being and resilience**

- - Depersonalisation (detachment) (older workers)^20^
  - Emotional exhaustion (older workers/total population)^20^
  - Optimisation (enhancement) (older workers/total population)^20^

1. **Workplace relationships**

**Leadership support**

- - Leadership visibility and supportive relationships^23^
  - Supportive and engaged leadership^25^
  - Engaged leadership^26^
  - Transparent communication and engagement^23^

| **Theme** | **Sub-theme** | **Code** | **Data quote** |
| --- | --- | --- | --- |
| Workplace relationships | Leadership support | Leadership visibility and supportive relationships | ‘We do tend to be people persons, and we pride ourselves on having good relationships with our staff as managers.’ ^23^  ‘We have an open-door policy here and I find that staff just fall in the office and blurt out whatever it is, or they’ll come in and shut the door and tell you, but I’m very visible.’^23^ |
|  |  | Supportive and engaged leadership | ‘Just [being] able to talk to [my boss about] situations… having someone to listen and understand.’^25^  ‘[Feeling] like their supervisor cares.’^25^  ‘Management to [be] visible on all the floors.’^25^ |
|  |  | Engaged leadership | ‘Willing to listen and help whenever needed and makes it very comfortable to talk about any problem with life or at work.’^26^  ‘I think that says a lot to people that work for you when you’ll get up out of your office chair and go out there and get your hands dirty with everybody else.’^26^  ‘If you make their job easier, they’ll work harder for you…making sure that you’re meeting the needs of your staff, basically, asking them what they need.’^26^ |
|  |  | Transparent communication and engagement | ‘Anything new we try; any new piece of equipment goes out on the floor and the staff will trial it for a month before I’ll look at buying it. Well, I don’t have to use it, they’ve got to use it, so they’ve got to say this works or this doesn’t work.’^23^  ‘The main thing is that you have an open forum with your staff and listen to them and certainly act on their concerns.’^23^ |

**Collaborative and supportive workplace relationships**

- - Positive workplace relationships^23^
  - Supportive relationships^24^
  - Fostering collaborative workplace relationships^25^
  - Formal and informal connections^30^

| **Theme** | **Sub-theme** | **Code** | **Data quote** |
| --- | --- | --- | --- |
| Workplace relationships | Collaborative and supportive workplace relationships | Positive workplace relationships | ‘I would say that the staff, like the people that you work with, are very supportive and friendly and the management is very supportive and you know if you’ve got any concerns, they’re very approachable.’^23^  ‘I think a lot of it is the relationships that are formed within the facility. Relationships with the residents, with other staff members, it becomes like an extended family and I think that’s mainly why people stay.’^23^  ‘The only thing is, that’s why I stay here longer, the management and then the nurses, you know, the other staff members that are nice, they are very good, you know.’^23^  ‘The more you know them the longer the friendships and relationships are getting closer.’ ^23^ |
|  |  | Supportive relationships | ‘The management, our managers, they were with us on the floors eh, they were there to help us.’^24^  ‘I remember the family, our manager’s wife, his daughter, they came eh, they were there to help us.’^24^  ‘Everyone came, everyone was there, and that gave us courage and strength.’^24^ |
|  |  | Fostering collaborative workplace relationships | ‘[Treated] like a big family and part of a team.’^25^  ‘Good working relationships [are] a game changer because if you do not like the people that you work with, you are more than likely going to be leaving the job sooner than later.’ ^25^ |

1. **Training and professional development**

**Specialist training programs**

- - Facility-based and non-facility-based training program/s^35^
  - Compassion fatigue awareness and self-care skills education program^34^
  - Nurse residency program^36^
  - Peer mentoring^33^

**Skill development and professional growth**

- - Career development and training opportunities^23^
  - Professional development and training opportunities^25^
  - Career progression^26^

| **Theme** | **Sub-theme** | **Code** | **Data quote** |
| --- | --- | --- | --- |
| Training and professional development | Skill development and professional growth | Career development and training opportunities | ‘The idea is to offer career pathways to our certificate 3s so that we not only develop them and they feel valued and they cannot get bored, but also so that we can develop skills and increase the workforce’s experience on the floor.’^23^  ‘We have our own regular education and we get people from outside to come in and give education.’ ^23^  ‘All of our programs are geared without saying, to staff retention, but we look at our education programs and scholarships to support staff through additional training.’^23^ |
|  |  | Professional development and training opportunities | ‘The staff here need better and more consistent training.’^25^  ‘Training staff to work all over the facility.’^25^ |
|  |  | Career progression | ‘When you see potential [in career pathways] and especially when your leaders see potential in you.’^26^  ‘Mutual benefit of promoting workers to become leaders and share their knowledge with the newer hires.’^26^ |

1. **Recognition, incentive and compensation**

**Employee recognition and appreciation**

- - Employee appreciation and recognition^25^
  - Recognising, valuing and rewarding employees^23^
  - Employee respect, recognition and appreciation^26^
  - Attendance awards^29^

| **Theme** | **Sub-theme** | **Code** | **Data quote** |
| --- | --- | --- | --- |
| Recognition, incentive and compensation | Employee recognition and appreciation | Employee appreciation and recognition | ‘If we don’t feel appreciated, we won’t stay.’^25^  ‘Recognition for a good job.’^25^  ‘Validation’ or ‘positive reinforcement.’^25^  ‘Wellness programs—chiropractor, massage for floor staff.’^25^  ‘Offer[ing] childcare services on-site or discounted rates because lots of CNAs are single moms and childcare is expensive.’^25^  ‘Others mentioned free snacks or a free meal…we throw away a lot of food here’ and ‘we’re making food anyway.’^25^ |
|  |  | Recognising, valuing and rewarding employees | ‘Probably every third month we have a pizza night or something and just give them a bit that way and we’re just about to do a reward recognition gift. We give a gift in the middle of the year and it’s just to make sure that they know what they do matters, even though there is no budget. I’d love to give each person $100 just because, but we can’t, so we give them a little trinket, something, a little saying, a little card.’^23^  ‘I just give movie passes basically, mentions in meetings is huge, going up and saying thank you, that sort of thing.’^23^  ‘They know I appreciate them and I get that back in spades, simple as that. They all get a card for their birthday and some scratchies [instant lottery tickets]. Nothing big, but they know that they’re appreciated.’^23^  ‘If we expect them to treat our residents with respect and dignity, as managers we need to treat our staff the same or they’re not going to project it: if we don’t project it, they won’t project it.’^23^  ‘Well apart from the care, we’re given respect and they treat us with respect ... It’s very client plus staff oriented. We’re just as important and it’s nice to be treated, you know, important.’^23^  ‘If people don’t feel that their role is important or their opinions aren’t valued, then they have no commitment.’^23^  ‘You have to pay more [for certificate 4s than 3s]. At the end of the day costs of recruiting and cost of agency [temporary] staff and cost of just turning over – just losing staff – it’s far better to have a slightly above-award staff, a paid and stable workforce, than to have to keep recruiting.’^23^ |
|  |  | Employee respect, recognition and appreciation | ‘You have to respect your employees that are already there and let them know that they’re still important and needed.’^26^  ‘This company is much more caring…. If you’re doing a good job, they’ll stick a little smiley face sticker in your mailbox.’^26^  ‘Every time that we have communication with them, we let them know that they’re appreciated…. It’s important for them to know that their job is probably one of the most important jobs here.’^26^  ‘Anybody who feels appreciated will always do more.’^26^ |

**Financial benefits**

- - LPN hourly starting wages (LPN)^12^
  - CNA hourly starting wages (RN, LPN)^12^
  - Health insurance^20^
  - Financial - increased fee schedule and on-call stipend^30^

1. **Organisational structure, culture and resources**

**Organisational structure and contextual dynamics**

- - Facility size^22^
  - Ownership^22^
  - Not-for-profit^2^
  - For-profit^27^
  - For-profit (CNA)^12^
  - Occupancy rate^27^
  - Average occupancy (RN, LPN, CNA)^12^
  - Unemployment rate of region (CNA)^12^

**Organisational resources and specialist roles**

- - Alzheimer’s unit^29^
  - Retention specialist^31^
  - Bedside electronic medical record^32^

**Adequate staffing levels**

- - RN hours per day per resident^27^
  - CNA hours per resident per day^27^
  - LPN hours per patient per day^27^
  - RN hours per patient per day (RN, CNA)^12^
  - LPN hours per patient per day (CNA)^12^
  - LPN overtime shifts in the last week (RN, CNA)^12^
  - Increased nursing staff^30^
  - Increased availability and role of registered staff^30^
  - On-call coverage^30^

**Workplace culture**

- - Supportive working conditions^26^

| **Theme** | **Sub-theme** | **Code** | **Data quote** |
| --- | --- | --- | --- |
| Organisational structure, culture and resources | Workplace culture | Supportive working conditions | ‘Improved communication would make things 10 times better, even if short-staffed.’^26^  ‘It’s not just them [agency staff] making more money than us, that kind of is like a slap in the face.’^26^  ‘I think the wages aren’t high enough here to attract somebody because there’s so much higher attraction out there…let’s be real.’^26^  ‘Family-like…our [care community] is very homelike…it’s very family oriented.’^26^ |

**Leadership continuity**

- - One NHA in the last year^27^
  - Two NHA in the last year^27^
  - Number of months DoN employed (RN, LPN, CNA)^12^
  - Director of nursing tenure in months^29^

1. **Values driven and empowered care practice**

**Commitment to person-centred care**

- - Fulfillment of resident care-needs^23^
  - Emotional commitment and moral obligation to caring^24^
  - Listening to resident stories^26^

| **Theme** | **Sub-theme** | **Code** | **Data quote** |
| --- | --- | --- | --- |
| Values driven and empowered care practice | Commitment to person-centred care | Emotional commitment and moral obligation to caring | ‘I felt guilty for staying at home.’ ^24^  ‘However, I was needed somewhere, it bothered me a bit, so that is a bit of what also helped my motivation to return to work.’ ^24^  ‘Can you imagine if all the CNAs say we are scared; who will care for the older people? First, it is a fact that the moral satisfaction of knowing that you are helping people who need it satisfies me.’ ^24^  ‘Some residents have practically no parents at all, so they take us as their parents, which satisfies me morally.’ ^24^ |
|  |  | Fulfillment of resident care-needs | ‘People are coming in later…but now they’re coming in with technical issues, very clinically technical issues, very clinically technical issues, which need to be dealt with, advanced dementias and the like.’^23^  ‘They’re just here for the money,’ and I said, ‘no, if they were here for the money, they’d be down in Franklins [supermarket] packing shelves and making more’, and not being abused and spat on and pooped on and all the rest that goes with aged care and especially with your dementias and your behaviours.’ ^23^ |
|  |  | Listening to resident stories | ‘One of my favourite things is you get to hear so many stories of these people’s lives that I would’ve never, ever even thought about happening to people.’^26^  ‘You just really get a connection on other people’s levels that I don’t think you would get at other jobs.’ ^26^ |

**Employee empowerment and role flexibility**

- - Staff empowerment practices^27^
  - Work latitude (autonomy) (older workers) ^20^
  - Enhancing job design and workforce adaptability^23^
  - Role flexibility^26^
  - Empowering working environment^25^

| **Theme** | **Sub-theme** | **Code** | **Data quote** |
| --- | --- | --- | --- |
| Values driven and empowered care practice | Employee empowerment and role flexibility | Enhancing job design and workforce adaptability | ‘I’ve redesigned her position to fit in with quality management. She won’t be getting a pay raise for it though. She loves that kind of work, she’s a systems person, you work with the talent that you’ve got.’^23^  ‘Even though there’s a no-lifting policy, still it can be hard work, so as I get older that would probably be a bit easier for me, you’re doing paper work and dressings, going towards that direction, that’s one of the reasons I did [Certificate 4] so that I could stay in aged care as I get a bit older.’ ^23^  ‘I believe we really need to look at how we’re going to meet the needs of our ageing workforce because they are going to become more prone to injury ... so we need to look at what equipment we need, what procedures, policies, processes we’re going to need to actually bypass some of these risk factors, ’cos otherwise we’ll be paying workers comp like nobody’s business.’ ^23^  ‘All of ours pretty much [work elsewhere]. They do a day shift here then they’ll do an afternoon shift somewhere else. Not too many are full time. They’re probably doing up to 30 hours [a week] here and then they might do a short shift somewhere else ... some of them work seven days a week and they’re 50, 60 years old.’ ^23^  ‘I have two jobs actually, I have to admit. So, Saturday, Sunday mornings I work somewhere else in another nursing home. Four days a week here and two days a week in another place.’ ^23^ |
|  |  | Role flexibility | ‘They want the freedom of picking what they want, when they want.’^26^  ‘We are trying to make them more independent in that and give them more freedom. They choose what order they do it.’^26^ |
|  |  | Empowering working environment | ‘[Mandating] deters people…mandating employees takes away from being able to have a personal life, especially with children.’^25^  ‘Not having flexible schedules can be difficult for single mothers.’ ^25^ |

**Abbreviations:** CNA: Certified nursing assistant; DoN: Director of nursing; LPN: Licensed practical nurse; NHA: Nursing home administrator; RN: Registered nurse.
