# Supplementary material for: A systematic, integrative review exploring supports that promote the retention of employees working in the aged care sector
Source: Australas J Ageing. 2025 Jul 31;44(3):e70070. doi: 10.1111/ajag.70070 (PMC12312298; doi:10.1111/ajag.70070)
Supplement: Supplementary file 3 — Appendix S3 [file AJAG-44-0-s005.docx]

**Appendix 3**

**Table 2:** Significant and non-significant findings from quantitative articles included in the review

| **Reference** | **Results** |
| --- | --- |
| Berridge et al (2018), USA | **Statistically significant**  The ordered logistic regression analysis revealed several significant positive associations between retention and factors such as staff empowerment quartile 2, (OR = 1.44, CI: 1.15, 1.80, *p* ≤ 0.01), staff empowerment quartiles 3 and 4 (OR = 1.64, CI: 1.34, 2.00, *p* ≤ 0.01), one NHA in the last year (OR = 1.768147, CI: 1.33, 2.35, *p* ≤ 0.01), two NHAs in the last year (OR = 1.410321, CI: 1.03, 1.92, *p* < 0.05), county unemployment rate (OR = 1.039485, CI: 1.00, 1.08, *p* < 0.05), occupancy rate (OR = 1.250274, CI: 1.14, 1.37, *p* ≤ 0.01), RN hours per day per resident (OR = 2.302827, CI: 1.59, 3.34, *p* ≤ 0.01) and CNA hours per day per resident (OR = 1.106825, CI: 1.01, 1.22, *p* < 0.05). For-profit status (OR = 0.7477272, CI: 0.62, 0.90, *p* ≤ 0.01) exhibited negative associations with retention.  **Not statistically significant**  The logistic regression analysis revealed that several variables were non- significantly associated with retention. These included facilities with <80 beds (OR = 1.137667, CI: 0.93, 1.39), facilities with >120 beds (OR = 1.166295, CI: 0.95, 1.43), chain affiliation (OR = 1.025854, CI: 0.86, 1.22), metropolitan county (OR = 1.10892, CI: 0.92, 1.33), residents with Medicaid (OR = 1.003315, CI: 0.99, 1.01), LPN hours per resident per day (OR = 0.7659152, CI: 0.57, 1.03, p < .10), county NH beds per 1,000 population 65+ (OR = 0.9992616, CI: 0.99, 1.00) and county home health agencies per 1,000 population 65+ (OR = 0.8831055, CI: 0.66, 1.18). |
| Chao and Lu (2020), Taiwan | **Statistically significant**  At T2, parameter estimates across the entire sample revealed significant positive associations between retention and marital status (OR = 11.974, (2.640, 54.316), *p* ≤ 0.001), optimisation strategies (OR = 3.352, (1.171, 9.597), *p* ≤ 0.05), and ITS (OR = 1.901 (1.066, 3.411), *p* ≤ 0.05).  While education of college and above (OR = 0.047 (0.002, 0.968), *p* ≤ 0.05), and emotional exhaustion (OR = 09.24, (0.854, 0.996), *p* ≤ 0.05) were negatively associated with retention.  For younger NAs (<45 years), at T2, significant positive associations were found between retention and gender (OR = 0.457 (0.223, 0.935), *p* ≤ 0.05), while negative associations were observed with marital status (OR = 2.544 (1.357, 4.737), *p* ≤ 0.01).  For younger NA (> 46 years) at T2, significant positive associations were identified between retention and marital status (OR = 1.853 (1.010, 3.400), *p* ≤ 0.05), work latitude (OR = 1.085 (1.000, 1.177), *p* ≤ 0.05) and optimisation strategies (OR = 1.959 (1.278, 3.003), *p* ≤ 0.01), while education level of college and above (OR = 0.260 (0.088, 0.775), *p* ≤ 0.05) and emotional exhaustion (OR = 0.959 (0.931, 0.987), *p* ≤ 0.01) were negatively association with retention.  **Not statistically significant**  At T2, parameter estimates combining the entire sample revealed the following variables, age (OR = 1.013 (10.941, 1.091)), gender (OR = 0.671 (0.098, 4.597)) education level - junior high school (OR = 0.204 (0.012, 3.507)) and senior high school (OR = 0.180 (0.012, 2.608)), income of 25,000 to 29,999 (OR = 2.371 (0.400, 14.072)) and 30,000 to 34,999 (OR = 4.995 (0.702, 35.534)), and 35,000 or above (OR = 4.964 (0.336, 73.351)), organisational support (OR = 0.760 (0.580, 1.013)), work latitude (OR = 1.196 (0.976, 1.465)), elective selection: prioritising personal goals (OR = 0.675 (0.329, 1.382)), loss-based selection: prioritising goals after losses (OR = 0.572 (0.253. 1.289)), compensation (OR = 0.710 (0.296, 1.702)), depersonalisation (OR = 1.080 (0.914, 1.277)) and personal accomplishment (OR = 0.991 (0.931, 1.056)), were not statistically significantly associated with retention.  Parameter estimates at T2 for younger NA (< 45 years) also revealed that variables such as education level - junior higher school (OR = 1.187 (0.061, 22.976)) and senior high school (OR = 1.033 (0.058, 18.514)), and college and above (OR = 0.997 (0.055, 18.217)), income of 25,000 to 29,999 (OR = 1.793 (0.896, 3.588)) and 30,000 to 34,999 (OR = 2.093 (0.936, 4.678)), and 35,000 or above (OR = 1.575 (0.477, 5.193)), organisational support (OR = 0.962 (0.865, 1.069)), work latitude (OR = 1.001 (0.923, 1.087)), selection: elective (OR = 0.881 (0.653, 1.190)), selection: loss-based (OR = 1.018 (0.733, 1.413)), optimisation (OR = 1.083 (0.722, 1.625)), compensation (OR = 0.796 (0.553, 1.146)), emotional exhaustion (OR = 0.992 (0.960, 1.025)), depersonalisation (OR = 0.947 (0.889, 1.008)), personal accomplishment (OR = 1.007 (0.981, 1.034)) and ITS (OR = 1.182 (0.931, 1.500)) were not statistically significantly associated with retention.  Parameter estimates at T2 for older NA (> 46 years) similarly indicated that variables such as gender (OR = 1.872 (0.851, 4.119)), education level - junior higher school (OR = 0.655 (0.232, 1.845)) and senior high school (OR = 1.033 (0.058, 18.514)), income of 25,000 to 29,999 (OR = 0.759 (0.342, 1.682)) and 30,000 to 34,999 (OR = 1.049 (0.459, 2.398)), and 35,000 or above (OR = 1.725 (0.553, 5.384)), organisational support (OR = 0.909 (0813, 1.015)), selection: elective (OR = 0.884 (0.649, 1.203)), selection: loss-based (OR = 0.720 (0.504, 1.029)), compensation (OR = 1.036 (0.715, 1500)), personal accomplishment (OR = 0.985 (0.957, 1.014)) and ITS (OR = 1.214 (0.971, 1.517)) were not statistically significantly associated with retention. |
| Dill et al (2013), USA | **Statistically significant**  In Regression analysis, specifically Model 5, it was found that retention was positively associated with availability of health insurance (Coef. 0.77, SE 0.37, *p* ≤ 0.05) tenure in the job (Coef. 0.47, SE 0.18, *p* ≤ 0.05) and negatively associated with being breadwinner (Coef. -0.92, SE 0.37, *p* ≤ 0.05), past health experience (Coef. -0.86, SE 0.35, *p* ≤ 0.05).  **Not statistically significant**  Regression analysis, specifically Model 5, revealed that several variables were not statistically significantly associated with retention. These included ITS (Coef. -0.12, SE 0.68), supervisor support (Coef. -0.50, SE 0.48), workload (Coef. -0.134 SE 0.50), financial rewards (Coef. -0.06, SE 0.26), career rewards (Coef. -0.09, SE 0.39), quality of coworkers (Coef. -0.63, SE 0.56), perceived quality of care (Coef. -0.76, SE 0.52), team care (Coef. 0.141 SE 0.49), being single mother (Coef. 0.93, SE 0.69), receiving public assistance (Coef. -0.35, SE 0.66), age (Coef. 0.09, SE 0.62), gender - female (Coef. -1.28, SE 0.98), black (Coef. -0.02, SE 0.31), another minority (Coef. 0.35, SE 0.96), high school degree (Coef. -0.77, SE 0.49), for-profit organisation (Coef. 0.31, SE 0.47), size/10 (Coef. 0.11, SE 0.08), unemployment rate (Coef. 0.06, SE 0.14) and comparable income (Coef. 0.21, SE 0.24). |
| Donoghue (2010), USA | **Statistically significant**  Weighted odds ratio estimates for RNs employed for 12 months or more indicated that retention was positively associated with the number of months the DoN was employed (OR = 0.12, CI: 0.02, *p* < 0.001), RN hours per patient per day (OR = 7.41, CI: 2.57, *p* < 0.01), CNA hourly starting wage (OR = 2.88, CI: 0.95, *p* < 0.01), and average occupancy (OR = 26.6, CI: 8.03, *p* < 0.01). Conversely, retention was negatively associated with LPN overtime shifts in the last week (OR = -0.35, CI: 0.16, *p* < 0.05).  Similarly, for LPNs employed for 12 months or more, retention was positively associated with the number of months the DoN was employed (OR = 0.09, CI: 0.02, *p* < 0.001), CNA hourly starting wages (OR = 2.22, CI: 0.81, *p* < 0.01), and average occupancy in the nursing home (OR = 2.32, CI: 7.89, *p* < 0.05). Conversely, LPN hourly starting wages were negatively associated with retention (OR = -2.08, CI: 0.58, *p* < 0.001).  For CNAs employed for 12 months or more, retention was positively associated with the number of months the DoN was employed (OR = 0.06, CI: 0.01, *p* < 0.001), RN hours per patient per day (OR = 8.20, CI: 2.67, *p* < 0.05), average occupancy in the nursing home (OR = 14.31, CI: 6.46, *p* < 0.05), and unemployment rate (OR = 0.97, CI: 0.34, *p* < 0.01). Conversely, LPN hours per patient per day (OR = -3.92, CI: 1.69, *p* < 0.05), LPN overtime shifts in the last week (OR = -0.21, CI: 0.09, *p* < 0.05), and for-profit nursing homes (OR = -4.34, CI: 1.65, *p* < 0.01) were negatively associated with retention.  **Not statistically significant**  The weighted odds ratio estimates for RNs employed 12 months or more did not reveal statistically significantly associated with retention for the following variables: number of months NHA employed (0.02, 0.01), LPN hours per patient day (-0.77, 2.14), CNA hours per patient day (0.11, 0.82), RN hours solely bedside per patient day (-0.99, 1.95), RN overtime shifts in the last week (0.33, 0.22), CNA overtime shifts in the last week (0.04, 0.07), RN hourly starting wages (hourly) (-0.35, 0.48), LPN hourly starting wages (-0.96, 0.62), bed size (-.00, .01), for-profit NHs (-1.96, 2.35), chain membership (-5.83, 2.33), Medicaid census (-.11, 4.91), per capita income (.02, .17), NHs in the county (.02, .02), unemployment rate (.60, .51).  Similarly, for LPNs employed 12 months or more, the following variables were not found to be statistically significantly associated with retention: number of months NHA employed (-0.01, 0.02), RN hours per patient day (2.86, 2.48), LPN hours per patient day (-2.02, 2.03), CNA hours per patient day (1.14, 0.71), RN hours solely bedside per patient day (0.72, 2.06), RN overtime shifts in the last week (0.12, 0.21), LPN overtime shifts in the last week (-0.20, 0.13), CNA overtime shifts in the last week (0.00, 0.05), RN hourly starting wages (hourly) (0.67, 0.42), bed size (-.01, .01), for-profit NHs (-1.49, 2.14), chain membership (-3.03, 2.06), Medicaid census (2.61, 4.72), per capita income (.23, .17), NHs in the county (-.02, .02), unemployment rate (.40, .43).  Lastly, for CNAs employed 12 months or more, retention did not exhibit statistically significantly associations with the number of months NHA employed (0.01, 0.01), CNA hours per patient day (0.91, 0.78), RN hours solely bedside per patient day (-4.57, 2.043), RN overtime shifts in the last week (0.09, 0.20), CNA overtime shifts in the last week (-0.02, 0.04), RN hourly starting wages (hourly) (0.33, 0.31), LPN hourly starting wages (-0.66, 0.44), CNA hourly starting wages (1.05, 0.72),bed size (.01, .01), chain membership (-.47, 1.63), Medicaid census (2.82, 3.50), per capita income (.26, .13), NHs in the county (-.01, .02), unemployment rate (.40, .43). |
| Dreher et al (2019), USA | **Statistically significant**  The study did not yield statistically significant results concerning retention.  **Not statistically significant**  Despite observing increased CNA retention and reduced reliance on supplemental agency staff, the study did not yield any statistical evidence of significant differences in retention rates following the intervention. |
| Frank et al (2006), Canada | **Statistically significant**  The study did not yield statistically significant results concerning retention.  **Not statistically significant**  Despite observing increased CNA retention and reduced reliance on supplemental agency staff, the study did not yield any statistical evidence of significant differences in retention rates following the intervention. |
| Hegeman et al (2007), USA | **Statistically significant**  Utilising various statistical analyses, including paired-samples t-test, repeated measure one-way ANOVA, lower-bound test, and LSD comparison, the investigation into the effectiveness of the ‘Growing Strong Roots’ peer mentoring program demonstrated several positive statistically significant associations. For Group 1, the three-month post-retention rate exhibited a significant increase (t = -2.572, df = 14, *p* < 0.02), while the six-month baseline rate was notably lower than both the three-month post-retention rate (t = -6.672, df = 14, *p* < 0.002) and the six-month post-retention rate (t = -3.672, df = 14, *p* < 0.03). Conversely, for Group 2, the three-month retention rate surpassed the six-month retention rate significantly (t = 4.080, df = 15, *p* < 0.001), and the six-month baseline rate was markedly lower than the three-month post-retention rate (t = -6.672, df = 14, *p* < 0.002), as well as the three-month baseline rate being lower than the three-month post-retention rate (t = -4.080, df = 15, *p* < 0.02).  Using paired samples t-test for the Peer Mentoring for Long-term Charge Nurses, the study revealed a statistically significant positive association with retention at the three-month post-test mark (t = -2.16 df = 12, *p* < 0.05).  **Not statistically significant**  For the wait-comparison group compared to the invention group who completed the ‘Growing Strong Roots’ peer mentoring program, from pre-test to post-test at 3 months, the study did not yield any statistically significant results concerning retention. |
| Hunt et al (2012), USA | **Statistically significant**  In Model 3 of the weighted multinomial logistic regression analysis, a negative association was found between attendance awards (OR = 0.570, CI: 0.390, 0.831), DoN tenure (OR = 0.974, CI: 0.967, 0.981) and presences of Alzheimer’s unit (OR = 0.669, CI: 0.467, 0.959) and employee retention.  **Not statistically significant**  In Model 3 of the weighted multinomial logistic regression analysis, no statistically significant associations with retention were found for the following variables in the comparison between High RN retention and Moderate RN retention: employee recognition (OR = 1.21 CI: 0.809, 1.81), conference reimbursement: (OR = 1.15, CI: 0.784, 1.70), career ladder (OR = 0.714, CI: 0.457, 1.11), tuition reimbursement (OR = 1.38, CI: 0.951, 2.01), career development (OR = 0.714 CI: 0.457, 1.11), retirement (OR = 1.18, CI: 0.833, 1.68), paid sick days (OR = 0.755, CI: 0.49, 1.16), paid personal day (OR = 0.789, CI: 0.558, 1.12), parenteral nutrition (OR = 1.09, CI: 0.808, 1.48), number of beds 3-99 (OR = 1.16, CI: 0.815, 1.64), Medicaid 0-59% (OR = 0.666, CI: 0.428, 1.04), Medicaid 60-79% (OR = 0.797, CI: 0.520, 1.22), Region – Northeast (OR = 1.03 CI: 0.641, 1.66), Region – South (OR = 0.690, CI: 0.467, 1.02), Region – West (OR = 0.863, CI: 0.536, 1.39), for profit (OR = 0.926, CI: 0.657, 1.31), chain affiliation (OR = 1.06, CI: 0.754, 1.48) and urban location (OR = 0.908, CI: 0.662, 1.25). |
| Kennedy et al (2020), USA | **Statistically significant**  Regression analysis found retention was positively associated with not-for-profit NHs (Coef. 3.30, SE: 1.64, *p* < 0.05).  **Not statistically significant**  Regression analysis revealed that several variables were not statistically significantly associated with retention including, chain affiliation (Coef. -1.20, SE: 1.49), government owned/operated (Coef. 2.04, SE: 6.67), number of beds (Coef. 0.39, SE: 0.22), presence of dementia/special care unit (Coef. 1.27, SE: 1.62), rural location (Coef. 2.74 SE: 1.77), occupancy rate (Coef. 0.07, SE: 0.08), % Medicaid paid (Coef. −0.007, SE: 0.09), % Medicare paid (Coef. −0.17, SE: 0.17), CNA hours per resident day (Coef. 1.52; SE: 1.34), LPN hours per resident day (Coef. −4.39, SE: 2.50), RN hours per resident day (Coef. −4.89, SE: 3.21), Activities staff hours per resident day (Coef. −2.58, SE: 8.13), LNHA turnover (two or more episodes in 2 years) (Coef. −2.89, SE: 1.73), DoN turnover (two or more episodes in 2 years) (Coef. −3.45, SE: 2.12), CNA consistent assignment (Coef. −0.23, SE: 1.65), CNA empowerment composite scale (Coef. 0.44, SE: 0.37), CNA hourly wage (Coef. 0.28, SE: 0.64), resident acuity (Coef. −0.85, SE: 0.78), % dementia or Alzheimer’s residents (Coef. 0.01, SE: 0.04), % psychiatric illness residents (Coef. −0.03, SE: 0.05), % intellectual disability residents (Coef. 0.38, SE: 0.30), unemployment rate (county) (Coef. −0.20, SE: 0.96). |
| Meyer et al (2012), USA | **Statistically significant**  The study did not yield statistically significant results concerning retention.  **Not statistically significant**  Descriptive statistics revealed the study did not find any statistically significant differences in retention related to demographic characteristics or the type of CNA training program attended (facility or non-facility-based training site). Additionally, no significant differences in training satisfaction between facility and non-facility-based training sites was reported. Overall, study did not yield statistically significant results concerning retention. |
| Pillemer et al (2008), USA | **Statistically significant**  In the within-group analysis conducted over time, the Rating of Quality Efforts to Retain Good Employees in the Facility indicated statistically significant findings at specific time points. Specifically, the Treatment (T) group demonstrated positive associations with retention from baseline to 6-month (mean value .876, *p* < 0.001) and when compared to the average of 6-month and 12-month (Mean value .516, *p* < 0.002). However, a negative association with retention was observed in the T group from 6-months to 12-months (mean value: -.720, *p* < 0.001). Additionally, the Treatment Effect group exhibited positive associations with retention from baseline to 6-month (*p* < 0.001) and when compared to the average of 6-month and 12-month (*p* < 0.009).  Similarly, utilising within-group analysis over time, the Positive Retention Effects Scale indicated statistically significant findings. Specifically, the comparison of the T group from baseline to 6-month revealed a positive association with retention (Mean value .488, *p* < 0.004), whereas the comparison from 6-month to 12-month showed a negative association with retention (mean value -.759, *p* < 0.001).  **Not statistically significant**  In the analysis conducted using within-group comparison over time, the Rating of Quality Efforts to Retain Good Employees in Facility did not yield statistically significant findings for the Control (C) group across various time points. Specifically, from baseline to 6-month (mean value -.007, *p* < 0.97), from baseline to 12-month (mean value -.207, *p* < 0.31), from 6-month to 12-month (mean value -.200, *p* < 0.30), and when comparing baseline to the average of 6-month and 12-month (mean value -.107, *p* < 0.53). Similarly, the T group did not show significant results from baseline to 12-month (mean value .156, *p* < 0.45), nor did the Treatment Effect group exhibit significance from baseline to 12-month (*p* < 0.21) and from 6-month to 12 month (*p* < 0.06).  Similarly, the analysis using the Positive Retention Effects Scale revealed non-significant findings for the C group across time points: baseline to 6-month (mean value .067, *p* < 0.71), baseline to 12-month (mean value -.086, *p* < 0.65), 6-month to 12-month (mean value -.153, *p* < 0.40), and baseline vs the average of 6-month and 12-month (mean value -.010, *p* < 0.95). The T group also displayed non-significant results from baseline to 12-month (mean value -.271, *p* < 0.16) and when comparing baseline versus the average of 6-month and 12-month (mean value -.108, *p* < 0.49).  Moreover, the Treatment Effect did not yield statistically significant findings across all time points: baseline to 6-month (*p* < 0.09), baseline to 12-month (*p* < 0.49), 6-month to 12-month (*p* < 0.02), and baseline vs the average of 6-month and 12-month (*p* < 0.60). |
| Rantz et al (2010), USA | **Statistically significant**  The study did not yield statistically significant results concerning retention.  **Not statistically significant**  A regression analysis revealed there was no discernible trend in the staff retention for any groups in the study over time following the implementation of the electronic medical record in the selected NHs. |
| Salmond et al (2017), USA | **Statistically significant**  The study did not yield statistically significant results concerning retention.  **Not statistically significant**  The nurse residency program achieved an 86% retention rate among participants in the first year, noting New Jersey's average RN retention rate of 54% at program start. |
| Singh and Schwab (1998), USA | **Statistically significant**  In Model III, multiple regression analysis revealed that several variables were positively and statistically significantly associated with the retention of nursing home administrators including, motivational commitment of administrator (Coef. 1.94, *p* ≤ 0.05), facility size (Coef. 0.03, *p* ≤ 0.01) and independent (stand-alone) ownership (Coef. 2.96, *p* ≤ 0.05).  **Not statistically significant**  In Model III, multiple regression analysis revealed that several variables, including hospital affiliation (Coef. -2.29) and intercept (Coef. -8.3), were not statistically significantly associated with administrator retention. |

**Abbreviations:** CNA: Certified nursing assistant; DoN: Director of nursing; ITS: Intention to stay; NA: Nursing assistant; NH: Nursing home; NHA: Nursing home administrator; RN: Registered nurse; T1: Time 1; T2: Time 2; USA: United States of America.
