# Supplementary material for: A systematic, integrative review exploring supports that promote the retention of employees working in the aged care sector
Source: Australas J Ageing. 2025 Jul 31;44(3):e70070. doi: 10.1111/ajag.70070 (PMC12312298; doi:10.1111/ajag.70070)
Supplement: Supplementary file 4 — Appendix S4 [file AJAG-44-0-s003.docx]

**Appendix 4**

**Table 1**: Definitions of employee retention

| **Author** | **Definition** | **Stated definition** |
| --- | --- | --- |
| Berridge et al (2018), USA | No | n/a |
| Chao and Lu (2020), Taiwan | No | n/a |
| Creapeau et al (2022), USA | No | n/a |
| Dill et al (2013), USA | No | n/a |
| Donoghue (2010), USA | Yes | Longer mean employment duration in the same facility. |
| Dreher et al (2019), USA | No | n/a |
| Frank et al (2006), USA | No | n/a |
| Hegeman et al (2007), USA | No | n/a |
| Hunt et al (2012), USA | No | n/a |
| Karmacharya (2023), USA | No | n/a |
| Kennedy et al (2020), USA | Yes | The % of staff still employed over a period of time. |
| Meyer et al (2012), USA | No | n/a |
| Mountford (2013), Australia | No | n/a |
| Pillemer et al (2008), USA | No | n/a |
| Rantz et al (2010), USA | No | n/a |
| Sabi Boun et al (2023), Canada | No | n/a |
| Salmond et al (2017), USA | No | n/a |
| Singh and Schwab (1998), USA | Yes | The length of employment of three years or longer at the same facility. |

Abbreviations: n/a: Not applicable
