# Supplementary material for: A systematic, integrative review exploring supports that promote the retention of employees working in the aged care sector
Source: Australas J Ageing. 2025 Jul 31;44(3):e70070. doi: 10.1111/ajag.70070 (PMC12312298; doi:10.1111/ajag.70070)
Supplement: Supplementary file 5 — Appendix S5 [file AJAG-44-0-s002.docx]

**Appendix 5**

**Table 1**: Mixed Methods Appraisal Tool (MMAT)^17^ methodological quality appraisal

|  |  |  |  |  |
| --- | --- | --- | --- | --- |
| Qualitative study design | **Creapeau et al (2022)** | **Karmacharya et al (2023)** | **Mountford**  **(2013)** | **Sabi Boun et al (2023)** |
| - 1. Is the qualitative approach appropriate to answer the research question?   2. Are the qualitative data collection methods adequate to address the research question?   3. Are the findings adequately derived from the data?   4. Is the interpretation of results sufficiently substantiated by data?   5. Is there coherence between qualitative data sources, collection, analysis and interpretation?   Total score | 1  1  1  1  1  **5** | 1  1  1  1  1  **5** | 1  1  1  1  1  **5** | 1  1  1  1  1  **5** |
| Quantitative randomized controlled design | **Pillemer et al**  **(2008)** |  |  |  |
| 2.1. Is the sampling strategy relevant to address the research question?  2.2. Is the sample representative of the target population?  2.3. Are the measures appropriate?  2.4. Is the risk of nonresponse bias low?  2.5. Is the statistical analysis appropriate to answer the research question?  Total score | 0  1  0  0  0  **1** |  |  |  |
| Quantitative non-randomized design | **Hegeman et al(2007)** | **Rantz et al**  **(2010)** |  |  |
| 3.1. Are the participants representative of the target population?  3.2. Are the measurements appropriate regarding both the outcome and intervention (or exposure)?  3.3. Are there complete outcome data?  3.4. Are the confounders accounted for in the design and analysis?  3.5. During the study period, is the intervention administered (or exposure occurred) as intended?  Total score | 0  1  1  0  0  **2** | 1  1  1  0  0  **3** |  |  |
| Quantitative descriptive study design | **Berridge et al**  **(2018)** | **Chao and Lu**  **(2020)** | **Dill et al**  **(2019)** | **Dreher et al**  **(2019)** |
| 4.1. Is the sampling strategy relevant to address the research question?  4.2. Is the sample representative of the target population?  4.3. Are the measurements appropriate?  4.4. Is the risk of nonresponse bias low?  4.5. Is the statistical analysis appropriate to answer the research question?  Total score | 1  0  1  1  1  **4** | 1  0  1  1  1  **4** | 1  0  1  0  1  **3** | 1  0  1  0  1  **3** |
| Quantitative descriptive study design | **Donoghue (2010)** | **Frank (2006)** | **Hunt et al**  **(2012)** | **Kennedy et al (2020)** |
| 4.1. Is the sampling strategy relevant to address the research question?  4.2. Is the sample representative of the target population?  4.3. Are the measurements appropriate?  4.4. Is the risk of nonresponse bias low?  4.5. Is the statistical analysis appropriate to answer the research question?  Total score | 1  1  1  1  1  **5** | 1  0  1  0  1  **3** | 1  1  1  0  1  **4** | 1  1  1  1  1  **5** |
| Quantitative descriptive study design | **Meyer et al**  **(2012)** | **Singh and Schwab (1998)** |  |  |
| 4.1. Is the sampling strategy relevant to address the research question?  4.2. Is the sample representative of the target population?  4.3. Are the measurements appropriate?  4.4. Is the risk of nonresponse bias low?  4.5. Is the statistical analysis appropriate to answer the research question?  Total score | 1  1  1  1  1  **5** | 1  0  1  1  1  **4** |  |  |
| Mixed methods study design | **Salmond et al (2017)** |  |  |  |
| 5.1 Is there an adequate rationale for using a mixed methods design to address the research question?  5.2 Are the different components of the study effectively integrated to answer the research question?  5.3 Are the outputs of the integration of qualitative and quantitative components adequately interpreted?  5.4 Are divergences and inconsistencies between quantitative and qualitative results adequately addressed?  5.5 Do the different components of the study adhere to the quality criteria of each tradition of the methods involved?  Total score | 1  1  1  0  1  **4** |  |  |  |

**Notes:** 1 = a score of ‘yes’; 0 = a score of ‘no’ or ‘can’t tell’

Mixed methods studies were given the score of their lowest scoring criterion, as recommended by MMAT.
